# Vacuum-Microwave vs. Hot-Air Drying of Valencia Orange Slices: Evaluation of Process Optimization and Drying Kinetics with Bioactive Bioaccessibility, Intestinal Barrier Function, Advanced Glycation End-Products, and Sustainability

**DOI:** 10.3390/foods15214013

**Published:** 2026-11-09

**Authors:** Neslihan Keçeli, Semih Latif İpek, Erva Parıldı, Osman Kola

**Affiliations:** 1 Department of Food Engineering, Faculty of Engineering, Institute of Graduate School, Adana Alparslan Türkeş Science and Technology University, 01250 Adana, Türkiye; nslhnozklkcl@gmail.com; 2 Department of Food Engineering, Faculty of Engineering, Adana Alparslan Türkeş Science and Technology University, 01250 Adana, Türkiye; slipek@atu.edu.tr (S.L.İ.); eparildi@atu.edu.tr (E.P.)

**Keywords:** vacuum microwave drying, hot air drying, response surface methodology (RSM), bioactive compound bioaccessibility, orange slices

## Abstract

This study comparatively optimized and evaluated the quality, bioaccessibility, intestinal integrity, advanced glycation end-products (AGEs), and sustainability metrics of Valencia orange slices using vacuum-microwave drying (VMD) and hot-air drying (HAD). Response surface methodology based on a Box–Behnken design established optimum conditions for VMD at 4 kW microwave power, 80 °C, and 2.228 mm slice thickness, and for HAD at 80 °C, 1.806 m/s air velocity, and 2.0 mm thickness. VMD demonstrated superior quality preservation, retaining significantly higher levels of bioactive phenolics, organic acids, and individual carotenoids compared to HAD. Following INFOGEST 2.0 in vitro digestion, VMD achieved a higher total phenolic bioaccessibility (~56% vs. 42%) and basolateral transport efficiency (~7.8% vs. 5.2%) while better preserving CaCo-2 intestinal barrier function (higher TEER and lower FITC-dextran permeability). Compared to HAD, VMD significantly reduced thermal degradation, resulting in lower hydroxymethylfurfural (~10 vs. ~41 mg/kg) and fluorescent AGEs (~180 vs. ~340 AU/g) (p<0.05). Kinetic evaluation revealed a sevenfold higher effective moisture diffusivity for VMD (1.07 × 10^−9^ m^2^/s) than HAD (1.49 × 10^−10^ m^2^/s). Multivariate analysis highlighted distinct separation between treatments based on drying kinetics, bioactive retention, and thermal markers. Thermodynamic and environmental analyses revealed an operational trade-off between the systems: while HAD exhibited a lower specific energy consumption ($2.32\pm 0.08{\;\mathrm{kWh}/\mathrm{kg}\;H}_{2}O$) and operational carbon emissions ($1.01{\;\mathrm{kg}\;\mathrm{CO}}_{2}{\text{-}\mathrm{eq}/\mathrm{kg}\;H}_{2}O$), VMD achieved a ~78% reduction in drying duration and superior preservation of bioactive compounds, despite its higher specific energy consumption ($4.12\pm 0.18{\;\mathrm{kWh}/\mathrm{kg}\;H}_{2}O$) and carbon emissions ($1.80{\;\mathrm{kg}\;\mathrm{CO}}_{2}{\text{-}\mathrm{eq}/\mathrm{kg}\;H}_{2}O$).

## 1. Introduction

The Valencia orange (*Citrus sinensis* L. Osbeck) is a commercially significant sweet orange variety due to its late maturity, extended harvest period, and the suitability of its fruit for both fresh consumption and industrial juice production [1,2]. Valencia oranges are rich sources of bioactive flavanones, carotenoids, and ascorbic acid, which offer strong antioxidant and chronic disease-preventive benefits; however, these compounds are highly susceptible to thermal and oxidative degradation during processing. Orange is a high-water-content fruit. There is a need to develop appropriate drying technologies to reduce post-harvest losses, extend shelf life, and transform the fruit into value-added products, such as dried slices [3]. The Valencia orange is a versatile raw material that can be utilized not only for juice and jam production but also in the development of dried fruit, natural flavoring agents, and snack products. Drying fruits that are difficult to market fresh or process can extend their usable life; it can also reduce the need for storage, packaging, and transportation [4]. Dried orange slices can be consumed directly or used as natural color and flavoring agents in herbal teas, beverages, bakery products, and various food formulations [5]. Furthermore, drying in sliced form allows for the utilization of the peel and albedo tissues alongside the fruit pulp. This provides the transformation of the orange into a value-added product available year-round and lessens the fresh fruit peel waste.

Drying removes water from fruit tissue, limiting microbial growth, enzymatic activity, and chemical degradation. In addition to extending shelf life, drying conditions—including temperature, duration, pressure, and energy input—can affect product color, structure, aroma, and rehydration capacity [6]. Orange slices contain albedo, peel, and pulp, which have different structural properties and can hinder uniform heat and moisture transfer [7]. Differences in moisture content, porosity, and resistance to moisture movement make slice thickness an important factor in drying time and product quality. Excessively harsh conditions may cause color changes, shrinkage, surface hardening, and reduced rehydration capacity, even when the target moisture level is reached [8].

Hot-air drying is widely used in the food industry because its equipment is relatively simple to operate and inexpensive [9]. In this method, convection transfers heat to the product surface, where water evaporates, while moisture moves from the product interior toward the surface [10]. This internal moisture transfer slows over time, extending drying and increasing energy use, especially for high-moisture fruits [11,12]. In orange slices, the peel and albedo further resist moisture movement [7]. Prolonged heating may also cause color darkening, aroma loss, shrinkage, surface hardening, and reduced rehydration capacity, while increasing specific energy consumption [4,13]. These limitations highlight the need to investigate alternative methods that remove moisture more quickly and controllably, with lower energy use, from Valencia orange slices.

Vacuum-microwave drying is based on the principle that microwave energy generates volumetric heat by interacting with polar molecules and ionic components within the product, while the vacuum environment lowers the boiling point of water [14,15]. The pressure difference between the vapor generated within the product by microwave energy and the drying environment can accelerate moisture transport from the interior to the surface, thereby reducing the internal moisture transfer resistance encountered in hot-air drying [15]. Studies conducted on orange slices demonstrate that VMD can increase drying rate and effective moisture diffusivity, shorten processing time, and improve rehydration capacity [4,6]. The drying performance of VMD depends not only on the total applied microwave power but also on the power density—defined as the microwave power applied per unit mass of the product; an increase in power density generally enhances the drying rate and reduces drying time [16,17]. However, high or improperly controlled microwave energy input can lead to non-uniform heating, the formation of hot spots, localized burning, and irregular moisture distribution in the final product due to the influence of the electromagnetic field, product geometry, moisture distribution, and dielectric properties [15,16]. Therefore, evaluating the relationship between microwave power, product load, process temperature, and slice thickness is crucial for limiting quality losses associated with overheating while achieving rapid drying and low specific energy consumption [18].

Drying performance and final product characteristics are determined by the individual and interactive effects of method-specific process variables, in addition to the drying technology employed. In the VMD process, microwave power, process temperature, and slice thickness are the primary variables influencing drying time, drying rate, and product characteristics; in the HAD process, these are air temperature, air velocity, and slice thickness. Investigating these factors through single-variable experiments may prove insufficient for elucidating the interactions and nonlinear relationships between them [19]. Response surface methodology enables the modeling of linear, interactive, and quadratic relationships between process variables and responses such as drying time, drying rate, color, and rehydration capacity [20]. The multi-response desirability approach applied to response models obtained via RSM enables the determination of optimum process conditions that strike an appropriate balance among all responses by combining the optimization targets set for each response into a single, unified desirability function [21]. In this study, VMD and HAD were optimized by utilizing independent experimental designs incorporating their respective process variables. Following the experimental validation of the determined optimum conditions, the energy requirements of the methods were compared by calculating the energy needed to remove a unit amount of water—expressed as specific energy consumption (SEC)—for both processes [22].

Investigating drying kinetics is essential for quantitatively describing the product’s moisture loss during the process, changes in drying rate, and the internal moisture transport mechanism. Fitting experimental moisture data to thin-layer drying models enables the identification of the model that best describes the drying behavior, as well as the determination of the model’s kinetic parameters. Evaluating model fit using statistical indicators—such as the determination coefficient (R^2^), root mean square error (RMSE) and reduced chi-square, allows for the comparison of the explanatory and predictive performance of different models. Furthermore, effective moisture diffusivity, calculated using Fick’s second law of diffusion, facilitates a quantitative assessment of internal moisture movement within Valencia orange slices [12,23]. Examining drying kinetics alongside energy indicators is significant for revealing how higher drying rates or effective moisture diffusivity impact specific energy consumption. However, there are limited studies in which VMD and HAD processes for Valencia orange slices are optimized using method-specific independent experimental designs and where the optimum conditions are compared in terms of drying kinetics, effective moisture diffusivity, specific energy consumption, color, and rehydration behavior [4,18]. The aim of this study is to optimize the process variables for drying Valencia orange slices using vacuum-microwave (VMD) and hot-air drying (HAD) methods and to comparatively evaluate the effects of these two systems on drying kinetics, energy requirements, and product properties. To this end, microwave power, process temperature, and slice thickness were evaluated as independent variables for the VMD process, while air temperature, air velocity, and slice thickness were assessed for the HAD process. Drying time, drying rate, color, and rehydration capacity were considered as the primary optimization responses, and optimal conditions for both methods were determined using response surface methodology (RSM) with a multi-response desirability approach. Experimental moisture data were fitted to thin-layer drying models to identify the best-fitting model and estimate effective moisture diffusivity. The energy requirements of VMD and HAD were compared using specific energy consumption, while power density was calculated to quantify microwave power per unit mass of product. This study separately optimized VMD and HAD for Valencia orange slices using process-specific experimental designs, then compared the optimal conditions in terms of drying kinetics, energy use, and quality. The findings are intended to clarify the balance between rapid moisture removal, low energy consumption, and retention of color and rehydration capacity, and to inform the development of industrial drying processes for Valencia oranges. To guarantee direct comparability during the kinetic, bioaccessibility, and quality assessment stages, optimized validation trials were conducted using standardized initial batch masses (~600 g), optimum slice thicknesses (~2.0 mm for HAD and ~2.2 mm for VMD), and target final moisture levels (<10% dry basis), with final water activity values consistently falling below a_w_ < 0.32.

## 2. Materials and Methods

### 2.1. Materials

Valencia oranges (*Citrus sinensis* L. Osbeck) were used in this study. The fruit was sourced from a commercial growing area in Adana City, Türkiye. To maintain postharvest quality, the oranges were stored at 4 °C during preparation and drying. Refrigerated storage was used to slow metabolic activity and thereby reduce quality deterioration. Analytical-grade reagents, including methanol, acetonitrile, acetic acid, and analytical standards (listed in Supplementary Table S1), were obtained from Merck (Darmstadt, Germany).

### 2.2. Sample Preparation

Sample preparation, storage, and drying by vacuum-microwave drying and hot-air drying were conducted according to the method described by Keçeli et al. (2026) [18]. Before drying, the oranges were cut into uniform slices to facilitate comparison of the effects of drying conditions on the resulting products. An industrial-grade Sirman 14251628 Mirra 250 Plus electric slicer (Sirman, Campodarsego, Italy) was used to prepare slices with thicknesses of 2, 4, and 6 mm, as specified in the experimental design. Following the slicing process, the samples were immediately subjected to either vacuum-microwave or hot-air treatment under the conditions determined by their respective Box–Behnken experimental designs [18]. While slicing protocols follow Keçeli et al. [18], all kinetic, bioaccessibility, Caco-2 barrier, AGEs, and exergy data represent entirely original measurements.

### 2.3. The Drying Systems

**Figure 1:**
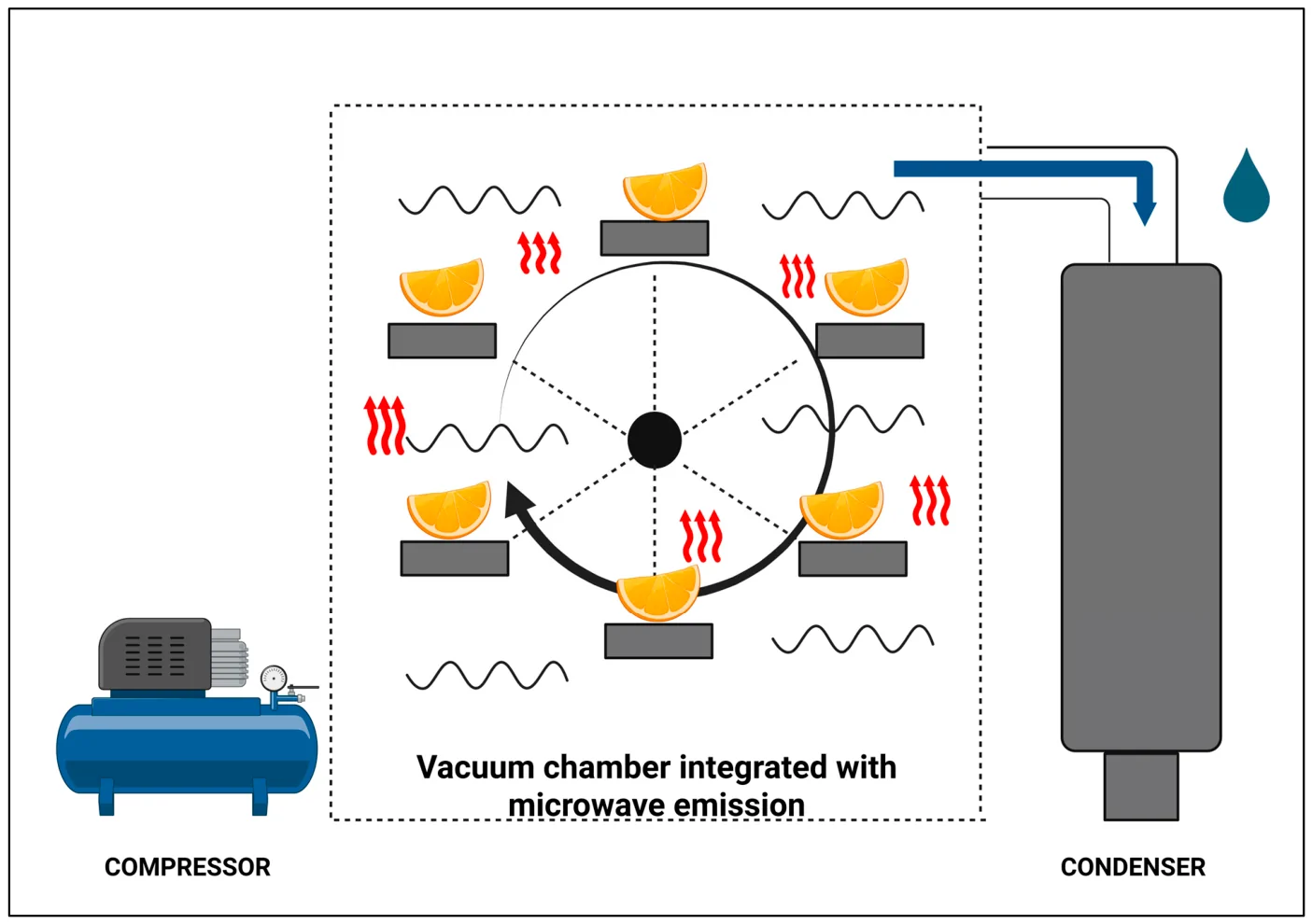
Schematic diagram of the vacuum-microwave dryer system (created with BioRender.com). The dashed lines indicate the structural partitions of the rotating tray mechanism, the curved black arrow shows the direction of rotation, and the red arrows represent the microwave radiation directed onto the orange samples.

Vacuum-microwave drying experiments were conducted using the prototype apparatus described by Bozkır (2020) [4]. The system was equipped with four high-frequency magnetrons, providing a power range of 1–4 kW to promote uniform energy distribution. During operation, the vacuum pressure varied with temperature, reaching approximately 199 mbar at 60 °C, 312 mbar at 70 °C, and 474 mbar at 80 °C. Orange slices were arranged in a single layer across five perforated Teflon trays (25 × 65 × 5 cm) to facilitate microwave absorption and moisture transfer. Sample mass was monitored using a precision balance until the target moisture content of less than 10% was reached, a level intended to support microbial stability and product shelf life (Figure 1).

A four-tier hot-air dryer (HAD; Adakurutma, Adana, Türkiye) with a stainless-steel frame was used for comparison. It had upper and lower heating elements and a fan that allowed horizontal airflow adjustment. Temperature and relative humidity were monitored through a digital interface. Orange slices were placed in a single layer on four perforated plastic trays (30 × 50 cm) and dried with hot air. Their mass was recorded regularly until the moisture content fell below 10%.

### 2.4. Optimization of Drying Conditions Using Response Surface Methodology (RSM)

The VMD and HAD processes were optimized using response surface methodology (RSM) via Design-Expert^®^ software (v13.0). For each drying method, a 3-factor, 3-level Box–Behnken design (BBD) comprising a physical experimental matrix of 17 runs (including 5 center points) was executed to model the process responses. Following model fitting and ANOVA validation, the multi-response desirability function approach was applied. A numerical search across the continuous model space generated a set of desirability-based optimization solutions, from which the optimal operating conditions with the highest overall desirability were selected and physically validated. The independent variable boundaries (e.g., maximum 4 kW microwave power, 80 °C temperature, and 2.0–2.2 mm slice thickness) were selected based on preliminary screening trials to prevent localized thermal scorching, volumetric charring, and equipment operational limits. It is acknowledged that the mathematical global optimum for certain kinetic parameters might extend beyond these tested upper boundaries; however, operating within these defined limits was necessary to ensure product quality integrity and avoid severe non-enzymatic thermal degradation.

### 2.5. Drying Kinetics and Mathematical Modeling

To characterize the drying curves of HAD and VMD, the experimental data were fitted to established thin-layer drying models [12]. Sample mass was recorded at 5 min intervals, and the moisture content of the orange slices at each time point was calculated using the following equation:

(1) $$Mt=\frac{W-W_{s}}{W_{s}}(kg\;H_{2}O/kg\;dry\;matter)$$

The total weight *W* (kg) is shown by the dry matter *W_s_* (kg).

The moisture ratio (MR) of the slices was determined using the following equation [24]:

(2) $$MR=\frac{M_{t}-M_{e}}{M_{0}-M_{e}}$$

*M_t_*: Moisture content of the slice at time t (kg H_2_O/kg dry matter).
*M*_0_: Initial moisture content (on a dry matter basis).
*M_e_*: equilibrium moisture content (on a dry matter basis).

The DR of the samples was determined via Equation (3):

(3) $$DR=\frac{\left(M_{t}-M_{t+dt}\right)}{d_{t}}$$

DR: kg H_2_O/kg dry matter·h.
*M_t_* and *M*_*t*+*dt*_: moisture content of the slice at times *t* and *t* + *dt* (kg H_2_O/kg dry matter).
t: Drying time (minutes).

MR is the dimensionless moisture ratio, and drying rate (DR, ${\mathrm{kg}\;H}_{2}O\cdot {\mathrm{kg}\;\mathrm{DM}}^{-1}\cdot h^{-1}$) is calculated using time intervals converted to hours.

Drying data for the orange slices were fitted to 10 commonly used thin-layer drying models. Model parameters were estimated by nonlinear regression to describe the moisture ratio as a function of drying time. Analyses were conducted using IBM SPSS v26.0. Model performance was evaluated using the coefficient of determination (R^2^), standard error of the mean, reduced chi-square (χ^2^), and root mean square error [25]. The best-fitting model was selected based on the highest R^2^ and the lowest χ^2^, RMSE, and SEM values. Effective moisture diffusivity was estimated from the slope of the linear relationship between ln and drying time, using the following equation:

(4) $$MR=\frac{8}{\pi^{2}}\sum_{0}\infty \frac{1}{\left(2n+1\right)}\exp\left[-\frac{\left({\left(2n+1\right)}^{2}\right)}{4}\;\frac{\pi^{2}D_{eff}t}{x_{1}^{2}}\right]$$

Simplified equation:

(5) $$LnMR=Ln\left(\frac{8}{\pi^{2}}\right)-\left(\frac{\pi^{2}D_{eff}t}{{4x}_{1}^{2}}\;\right)\;$$

*D_eff_*: m^2^/s.
*x*_1_: Represents the half-thickness (m) of sample.

Linear regression was performed using SPSS v26.0 to determine the slope (K) from the relationship between the experimental data and the theoretical model:

(6) $$K=\frac{\pi^{2}D_{eff}}{{4x}_{1}^{2}}$$

Within the array of thin-layer drying equations cited in the literature, 10 frequently employed empirical and semi-empirical models were identified after an initial review to represent fruit-drying behavior [26,27]. The best-fitting model was selected based on a strict decision hierarchy: primary priority was given to the highest coefficient of determination ($R^{2}$), followed sequentially by the lowest reduced chi-square ($\chi^{2}$) and root mean square error (RMSE) values.

### 2.6. Physicochemical and Rehydration Properties

Dried samples were ground using an analytical mill IKA A 10 basic, (IKA-Werke GmbH & Co. KG, Staufen, Germany). Moisture content and dry matter were determined by drying the samples to constant weight at 105±1 °C in an atmospheric drying oven according to the standardized gravimetric method (AOAC Official Method 935.29) [28]. Water activity (a_w_) was measured at 20 ± 1 °C. Titratable acidity was determined by titration with 0.1 N NaOH to pH 8.1 (AOAC 942.15) and expressed as citric acid percentage. Total soluble solids (°Brix) and pH were measured at 20 ± 1 °C using a digital refractometer and a calibrated pH meter, respectively.

Surface color coordinates (L*, a* and b*) were measured using a Konica Minolta CM-5 spectrophotometer with a D65 illuminant and 10° observer. Chroma (C*), hue angle (h°), and total color difference (ΔE) relative to fresh slices were calculated from these measurements. Rehydration capacity was determined following the method of Cui et al. (2008) [29]: A 10 g portion of dried sample was immersed in 200 mL of ultrapure water at 25 ± 1 °C for 24 h. The sample was then filtered, and the rehydrated mass (W_r_) was measured relative to the initial dry mass (W_d_). The formula is as follows:

(7) $$Rehydration\;capacity=\frac{W_{r}}{W_{d}}$$

W_r_: Product weight after rehydration (g).
W_d_: Initial dry weight of the dried product (g).

### 2.7. HPLC-PDA Profiling of Individual Bioactive Compounds

Targeted monomeric phenolic compounds, carotenoids, organic acids, and hydroxymethylfurfural were quantified using an HPLC–PDA system (Prominence-i LC-2030C 3D Plus, Shimadzu, Japan), following optimized methods reported in the literature.

Phenolic profile: Powdered samples (0.50 g) were extracted with methanol (1:10, *w*/*v*) at 4 °C for 24 h and filtered through 0.45 µm membranes. Compounds were separated on an Inertsil ODS-3 C18 column (5 µm, 250 × 4.6 mm) at 25 °C using a gradient of 3% aqueous acetic acid (A) and acetic acid–acetonitrile–water (3:25:72, *v*/*v*/*v*; B). The flow rate was 1.0–1.2 mL/min, and the injection volume was 20 µL. Detection was performed at 280, 320, and 360 nm. Compounds were quantified using 24 external standards [30].

Carotenoid profile: Residues were extracted with methanol:n-hexane containing 0.1% BHT and redissolved in acetonitrile. Carotenoids were separated on an Inertsil ODS-3 column at 25 °C using an acetonitrile–methanol–ethyl acetate gradient at 0.7 mL/min. PDA detection was performed at 450 nm to quantify six carotenoids: α-carotene, β-carotene, α-cryptoxanthin, β-cryptoxanthin, lutein, and zeaxanthin [31].

Organic Acids and HMF: Ascorbic, citric, malic, tartaric, and succinic acids were extracted with ultrapure water and separated isocratically on a Coregel ORH 801 column (300 × 6.5 mm) at 45 °C. The mobile phase was 0.01 N H_2_SO_4_, delivered at 0.4 mL/min. Detection was performed at 210 nm, except for ascorbic acid, which was detected at 244 nm. HMF was extracted with water and analyzed on a Capcell Pack C18 MGII column at 40 °C. Methanol–water (10:90, *v*/*v*) was used as the mobile phase at 1.0 mL/min, with detection at 285 nm. Quantification of organic acids was facilitated by comparing retention times and UV spectra against established analytical standards [32,33].

### 2.8. Chromatographic Carbohydrate Analysis (HPLC-RID)

Individual soluble sugars (glucose, fructose, sucrose, and sorbitol) were quantified using HPLC with a refractive index detector. Aqueous extracts (1.00 g sample/10 mL) were filtered through 0.2 µm membranes, and 20 µL aliquots were injected onto a Concise Separation CarboSep CHO-87 column. Isocratic separation was performed with ultrapure water at a flow rate of 0.6 mL/min and a column temperature of 84 °C. Sugars were identified and quantified using external calibration curves prepared from the corresponding standards. Results were expressed as mg/L [32].

### 2.9. In Vitro Static Gastrointestinal Digestion and Bioaccessibility Assessment

Simulated static gastrointestinal digestion of fresh and dried Valencia orange slices was performed according to the INFOGEST 2.0 protocol [34]. Digestion included oral, gastric, and intestinal phases under controlled conditions. For the oral phase, 1.25 g of pulverized sample was mixed 1:1 (*w*/*v*) with Simulated Salivary Fluid (SSF; pH 7.0) containing human salivary α-amylase (75 U/mL) and incubated at 37 °C for 2 min. The resulting bolus was mixed with Simulated Gastric Fluid (SGF; pH 3.0) and porcine pepsin (final activity, 2000 U/mL), then incubated at 37 °C for 2 h with orbital shaking at 90 rpm. For the intestinal phase, the gastric chyme was mixed with Simulated Intestinal Fluid (SIF; pH 7.0), pancreatin (100 U/mL trypsin activity), and fresh bile salts (final concentration, 10 mM), then incubated at 37 °C for 2 h. Enzymatic activity was stopped by heating the samples at 85 °C for 10 min. The digesta were centrifuged at 10,000× *g* for 20 min at 4 °C. Supernatants were filtered through 0.45 µm membranes and stored at −80 °C until analysis of total phenolic content, according to [18,35]. Digestion blanks (enzyme and bile salts without sample) were run in parallel to subtract baseline interferences. Total phenolic content was measured at 765 nm via the Folin–Ciocalteu method [35].

The Bioaccessibility Index (BI,%) was determined using Equation (8):

(8) $$\mathrm{BI}\;\left(\%\right)=\left(\frac{C_{\mathrm{digested}}\times V_{\mathrm{final}}}{C_{\mathrm{undigested}}\times m_{\mathrm{sample}}}\right)\times 100$$

where $C_{\mathrm{digested}}$ is the bioactive concentration in the intestinal digesta (mg/mL), $V_{\mathrm{final}}$ is the total volume of the intestinal digesta (mL), $C_{\mathrm{undigested}}$ is the bioactive concentration in the initial dried sample (mg/g dry matter), and $m_{\mathrm{sample}}$ is the initial dry sample mass (g).

Total phenolic content (TPC) was determined spectrophotometrically at 765 nm using the Folin–Ciocalteu assay and expressed as mg gallic acid equivalents (GAE)/100 g dry matter [35]. Total carotenoid content was quantified by measuring absorbance at 450 nm following extraction with a hexane:acetone:ethanol mixture, while DPPH radical scavenging capacity was evaluated at 517 nm to determine total antioxidant activity [36].

### 2.10. Caco-2 Cell Culture, Cytotoxicity (MTT and Neutral Red Assay), Epithelial Barrier Integrity (TEER), and FITC-Dextran Permeability

Caco-2 human colon adenocarcinoma cells were cultured in DMEM supplemented with 10% (*v*/*v*) fetal bovine serum, 1% (*v*/*v*) non-essential amino acids, 100 U/mL penicillin, and 100 µg/mL streptomycin at 37 °C in a humidified atmosphere with 5% CO_2_. Before conducting transport studies, the cytotoxicity of intestinal digesta on Caco-2 cells was evaluated using both MTT and Neutral Red uptake assays [37,38,39,40,41]. Cells were seeded in 96-well plates at a density of 1 × 10^4^ cells/well and cultured for 24 h. They were then exposed for 24 h to serial dilutions of intestinal digesta (1:5–1:100, *v*/*v*, in serum-free DMEM); medium alone was used as the control. For the MTT assay, MTT solution (20 µL; 5 mg/mL in PBS) was added to each well, and the plates were incubated at 37 °C for 4 h. The resulting formazan crystals were dissolved in 150 µL of DMSO, and absorbance was measured at 570 nm using a microplate reader. For the Neutral Red assay, the exposure medium was replaced with Neutral Red dye solution (40 µg/mL in DMEM) and incubated for 2 h at 37 °C. After washing with PBS, the internalized dye was extracted using a solubilization solution (50% ethanol, 49% deionized water, and 1% glacial acetic acid), and absorbance was measured at 540 nm. Digesta dilutions maintaining cell viability above 90% in both assays were considered non-cytotoxic and selected for subsequent epithelial barrier assays. For transport and barrier integrity experiments, cells were seeded onto polycarbonate Transwell membrane inserts (0.4 µm pore size, 12 mm diameter, Corning Costar) at a density of 1 × 10^5^ cells/cm^2^ and cultured for 21 days to achieve complete differentiation and tight junction formation. Monolayer barrier integrity was evaluated before and after exposure to non-toxic intestinal digesta dilutions by measuring transepithelial electrical resistance (TEER) using a digital volt–ohm meter equipped with a chopstick electrode (Millicell ERS-2, Millipore, USA), wherein monolayers exhibiting initial TEER values above 500 Ω·cm^2^ were accepted for permeability testing [42]. Paracellular permeability was measured using a fluorescein isothiocyanate–dextran (FITC-dextran, 4 kDa; FD4; Sigma-Aldrich) flux assay. Differentiated Caco-2 monolayers were rinsed twice with pre-warmed Hanks’ Balanced Salt Solution (HBSS; 10 mM HEPES, pH 7.4). The apical chamber received 0.5 mL of non-cytotoxic intestinal digesta dilution or HBSS control containing 1 mg/mL FITC-dextran. The basolateral chamber received 1.5 mL of HBSS. After incubation at 37 °C for 120 min, 100 µL samples were collected from the basolateral chamber. Fluorescence was measured at excitation and emission wavelengths of 485 and 530 nm, respectively [43]. The apparent permeability coefficient (*P*_app_, cm/s) was calculated using the equation below:

(9) $$P_{\mathrm{app}}=\frac{dQ}{dt}\times \frac{1}{A\cdot C_{0}}$$

where *dQ*/*dt* is the rate of FITC-dextran transport across the monolayer (µg/s), *A* is the surface area of the membrane insert (cm^2^), and *C*_0_ is the initial concentration in the apical chamber (µg/mL). The basolateral transport efficiency of total phenolics across the Caco-2 monolayer (%) was determined by measuring the total phenolic content in the basolateral chamber after 120 min incubation relative to the initial apical loading amount [44]. TEER values were measured after 120 min incubation (n=3 biological replicates) and normalized to initial baseline values using HBSS and digestion blanks.

### 2.11. Determination of Total Fluorescent Advanced Glycation End-Products (AGEs)

Total fluorescent advanced glycation end-product-like compounds formation was measured fluorometrically using the method of Li et al. [45], with minor modifications. Fresh and dried orange powders (0.50 g) were extracted with PBS (10 mL, 0.01 M, and pH 7.4) by shaking at 150 rpm for 2 h at room temperature (25 ± 1 °C). The suspensions were centrifuged at 8.000× *g* for 15 min at 4 °C, and the supernatants were filtered through 0.45 µm nylon syringe filters. Fluorescence was measured using a microplate fluorescence spectrophotometer at excitation and emission wavelengths of 370 and 440 nm, respectively. PBS was used as the blank. Fluorescent AGE-associated fluorescence was expressed as arbitrary fluorescence units per gram of dry matter (AU/g dry matter).

### 2.12. Specific Energy Consumption and Power Density Calculation for Drying Systems

Specific energy consumption was calculated using nominal power input. For VMD, this was the microwave power; for HAD, it included the heating components and fan, following previously reported methods [46,47]. The data used for these calculations are provided in Supplementary Table S3. SEC was calculated as follows:

(10) $$SEC=\frac{P\times t}{m_{ev}}$$

*SEC* denotes specific energy consumption (kWh/kg water evaporated), *P* is the nominal power input (kW), *t* is the drying time (h), and *m_ev_* is the mass of evaporated water (kg). Power density (W/g) was calculated as the microwave power divided by the initial mass of the orange sample in each trial. The specific energy consumption (SEC, $\mathrm{kWh}\cdot {\mathrm{kg}}^{-1}{\;H}_{2}O$) was evaluated based on the total operational electrical energy input (P), which encompasses nominal magnetron emission power and conversion losses for VMD, continuous vacuum pump operation, and thermostat-controlled heating element cycling for HAD.

### 2.13. Energy, Exergy, Sustainability, and Environmental Performance Analysis

To evaluate the operational energy requirements and provide a preliminary, evaporation-specific exergy indicator for the VMD process under optimized conditions (microwave power: 4 kW; drying temperature: 80 °C; slice thickness: 2.2 mm; operating chamber absolute pressure: 47.4 kPa/474 mbar; drying time: 35 min), a simplified thermodynamic and environmental assessment framework was applied. This evaluation is restricted to primary energy utilization and latent heat evaporation exergy, rather than a comprehensive system-level exergy closure incorporating physical and chemical exergies of all inlet/outlet stream flows [48].

### 2.14. Energy Metrics and Energy Utilization Efficiency

The total nominal electrical energy input ($E_{\mathrm{input}}$, kWh/batch or MJ/batch) during the drying process was evaluated based on the nominal power consumption and processing duration [49]. The specific energy consumption (SEC, $\mathrm{kWh}\cdot {\mathrm{kg}}^{-1}{\;H}_{2}O$ or $\mathrm{MJ}\cdot {\mathrm{kg}}^{-1}{\;H}_{2}O$) and the specific moisture extraction rate (SMER, ${\mathrm{kg}\;H}_{2}O\cdot {\mathrm{kWh}}^{-1}$) were calculated as follows:

(11) $$\mathrm{SEC}=\frac{E_{\mathrm{input}}}{m_{w}}$$

(12) $$\mathrm{SMER}=\frac{m_{w}}{E_{\mathrm{input}}}$$

where $m_{w}$ is the total mass of moisture removed per batch (${\mathrm{kg}\;H}_{2}O$).

The energy utilization efficiency ($\eta_{\mathrm{en}}$, %) was determined by comparing the latent heat required for moisture evaporation ($Q_{\mathrm{evap}}$, MJ/batch) to the total nominal energy input ($E_{\mathrm{input}}$) [50]:

(13) $$\eta_{\mathrm{en}}=\left(\frac{Q_{\mathrm{evap}}}{E_{\mathrm{input}}}\right)\times 100$$

(14) $$Q_{\mathrm{evap}}=m_{w}\cdot h_{\mathrm{fg}}$$

where $h_{\mathrm{fg}}$ is the latent heat of vaporization of water evaluated at the average product temperature ($T_{p}=40$ °C) [51]. Product temperature ($T_{p}=40$ °C) reflects the evaporative cooling stage, and $c_{p}$ denotes the specific heat capacity of the fruit matrix.

### 2.15. Simplified Exergy and Sustainability Analysis

The exergy associated with moisture evaporation (${\mathrm{Ex}}_{\mathrm{evap}}$, kJ/batch) was calculated based on the latent heat of evaporation, the product temperature ($T_{p}=40\;{}^{\circ}C/313.15\;K$), and the reference dead-state temperature ($T_{0}=25\;{}^{\circ}C/298.15\;K$) [48]:

(15) $${\mathrm{Ex}}_{\mathrm{evap}}=Q_{\mathrm{evap}}\cdot \left(1-\frac{T_{0}}{T_{p}}\right)$$

The evaporation-based exergy efficiency ($\eta_{\mathrm{ex}}$, %) was expressed as [52,53]:

(16) $$\eta_{\mathrm{ex}}=\left(\frac{{\mathrm{Ex}}_{\mathrm{evap}}}{E_{\mathrm{input}}}\right)\times 100$$

Note: The Carnot quality factor $\left(1-T_{0}/T_{p}\right)$ for moisture evaporation at $T_{p}=40$ °C (313.15 K) relative to $T_{0}=25$ °C (298.15 K) equals 0.0479. Consequently, comparing the low thermal exergy of water evaporation (${\mathrm{Ex}}_{\mathrm{evap}}=58.19\;\mathrm{kJ}/\mathrm{batch}$) directly against total nominal electrical energy input ($E_{\mathrm{input}}=8400\;\mathrm{kJ}/\mathrm{batch}$) mathematically yields an evaporation-based exergy efficiency of 0.693%. This value accurately reflects the operational thermodynamic quality loss inherent when high-grade electrical energy is utilized for low-temperature phase change in standalone thin-layer drying.

Within this simplified framework, an apparent unaccounted exergy term (${\mathrm{Ex}}_{\mathrm{unaccounted}}$, MJ/batch) was defined as the difference between nominal electrical energy input and evaporation exergy, rather than total system exergy destruction, due to unquantified inlet/outlet physical and chemical exergy streams [48,53]:

(17) $${\mathrm{Ex}}_{\mathrm{unaccounted}}=E_{\mathrm{input}}-{\mathrm{Ex}}_{\mathrm{evap}}$$

The simplified exergetic sustainability index (${\mathrm{SI}}_{\mathrm{ex}}$) was subsequently evaluated using the exergy efficiency [53,54]:

(18) $${\mathrm{SI}}_{\mathrm{ex}}=\frac{1}{1-\eta_{\mathrm{ex}}}$$

Note: Since the operational chamber pressure (47.4 kPa) corresponds closely to the saturation vapor pressure of water at 80 °C, non-condensable gas pressure contributions were neglected in the simplified exergy calculations.

### 2.16. Environmental Assessment (Operational Greenhouse-Gas Emissions)

Operational greenhouse-gas (GHG) emissions associated with electricity consumption during drying were estimated using a regional grid electricity emission factor (${\mathrm{EF}}_{\mathrm{grid}}=0.436{\;\mathrm{kg}\;\mathrm{CO}}_{2}\text{-}\mathrm{eq}\cdot {\mathrm{kWh}}^{-1}$) [55].

The total equivalent ${\mathrm{CO}}_{2}$ emissions per batch ($E_{{\mathrm{CO}}_{2},\mathrm{batch}}$, ${\mathrm{kg}\;\mathrm{CO}}_{2}\text{-}\mathrm{eq}/\mathrm{batch}$) and specific emissions per unit of evaporated water ($E_{{\mathrm{CO}}_{2},\mathrm{water}}$, ${\mathrm{kg}\;\mathrm{CO}}_{2}\text{-}\mathrm{eq}\cdot {\mathrm{kg}}^{-1}{\;H}_{2}O$) were calculated as follows:

(19) $$E_{{\mathrm{CO}}_{2},\mathrm{batch}}=E_{\mathrm{input}}\cdot {\mathrm{EF}}_{\mathrm{grid}}$$

(20) $$E_{{\mathrm{CO}}_{2},\mathrm{water}}=\frac{E_{{\mathrm{CO}}_{2},\mathrm{batch}}}{m_{w}}$$

### 2.17. Statistical Analysis, PCA, and Pearson Correlation

All experiments and analytical measurements were performed in triplicate. Results are reported as mean ± standard deviation. Differences among experimental conditions were assessed by one-way analysis of variance, followed by Tukey’s post hoc test. Statistical significance was set at *p* < 0.05. Analyses were conducted using SPSS v26.0 (IBM Corp., Armonk, NY, USA) and OriginPro 2018 (Version 9.5, OriginLab Corporation, Northampton, MA, USA). To examine multivariate relationships and distinguish samples from VMD and HAD treatments, principal component analysis was performed on standardized data. Pearson correlation analysis was used to assess linear associations among process variables (power, temperature, and slice thickness) and primary optimization response attributes (drying rate, rehydration capacity, and color coordinates). Statistical significance was set at *p* < 0.05. Model fit and predictive performance were evaluated using analysis of variance, the coefficient of determination (R^2^ and adjusted R^2^), residual diagnostics, and three-dimensional response-surface plots.

## 3. Results and Discussion

### 3.1. Optimization of Drying Parameters in VMD and HAD Systems for Valencia Orange Slices Using the Response Surface Methodology (RSM)

The effects of microwave power, drying temperature, and slice thickness on the drying of Valencia orange slices were evaluated using a vacuum-microwave dryer. Seventeen trials were conducted using a Box–Behnken design within the response surface methodology framework. Drying rate (DR; kg H_2_O/kg dry matter·h), rehydration capacity (g/g), and b* color value were measured as response variables. The experimental conditions and results are shown in Table 1. Factor coding followed standard normalization $X_{i}=\left(x_{i}-x_{0}\right)/\Delta x$, mapping factor levels to −1,0, +1. Model equations are presented in both coded and actual engineering units. Drying trials were strictly standardized across systems using ∼600 g initial load, 2–4 kW microwave power range, and target final $a_{w}<0.32$.

Using the acquired data, response surface methodology was applied to optimize the drying conditions. Second-order polynomial models were fitted to the coded independent variables for drying rate, rehydration capacity, and b* value. The resulting equations are presented as Equations (8)–(10). These models describe the relationships between the drying parameters and the measured responses and were used to predict response values:

DR = +4.60 + 0.6078 A + 0.7768 B − 3.07 C + 0.0524 AB − 0.0670 AC − 0.3664 BC + 0.3205 A^2^ − 0.1450 B^2^ + 1.66 C^2^ (21)

Rehydration Capacity = +4.12 − 0.0993 A − 0.0981 B − 0.4360 C + 0.0807 AB + 0.1298 AC − 0.2460 BC + 0.0107 A^2^ + 0.1384 B^2^ + 0.1630 C^2^ (22)

b* value = +36.69 + 1.22 A + 0.7108 B + 5.47 C − 1.84 AB − 1.04 AC − 0.4400 BC + 2.94 A^2^ + 0.7677 B^2^ − 1.55 C^2^ (23)

Independent Variables; A = Power (kW), B = Temperature (°C), C = Slice Thickness (mm).

ANOVA results showed that the second-order models fit the data well. The R^2^ values were 0.9970 for drying rate, 0.9728 for rehydration capacity, and 0.9729 for b* value, indicating that the models explained most of the variation in these responses (Table S4). All models were statistically significant (*p* ≤ 0.0001). Their coefficients of variation were low (2.21–3.69%), and their F-values ranged from 27.86 to 262.33. The lack-of-fit tests were not significant (*p* > 0.05), supporting the adequacy of the models. Residual plots also showed that residuals were approximately normal and randomly distributed for the representative DR responses in both drying systems (Figures S1 and S2). The regression plots (Figures S3–S5) also show good agreement between predicted and experimental values, supporting the model’s predictive performance. Although the lack-of-fit test for the VMD rate model yielded a boundary p-value (p=0.0514, F=6.48), it remains statistically non-significant (p>0.05), confirming adequate model fit. This slight elevation above the threshold reflects inherent operational noise associated with rapid localized vapor generation during vacuum-microwave heating rather than structural model inadequacy, as further evidenced by the exceptionally high $R^{2}$ (0.9970) and low coefficient of variation (3.69%).

**Figure 2:**
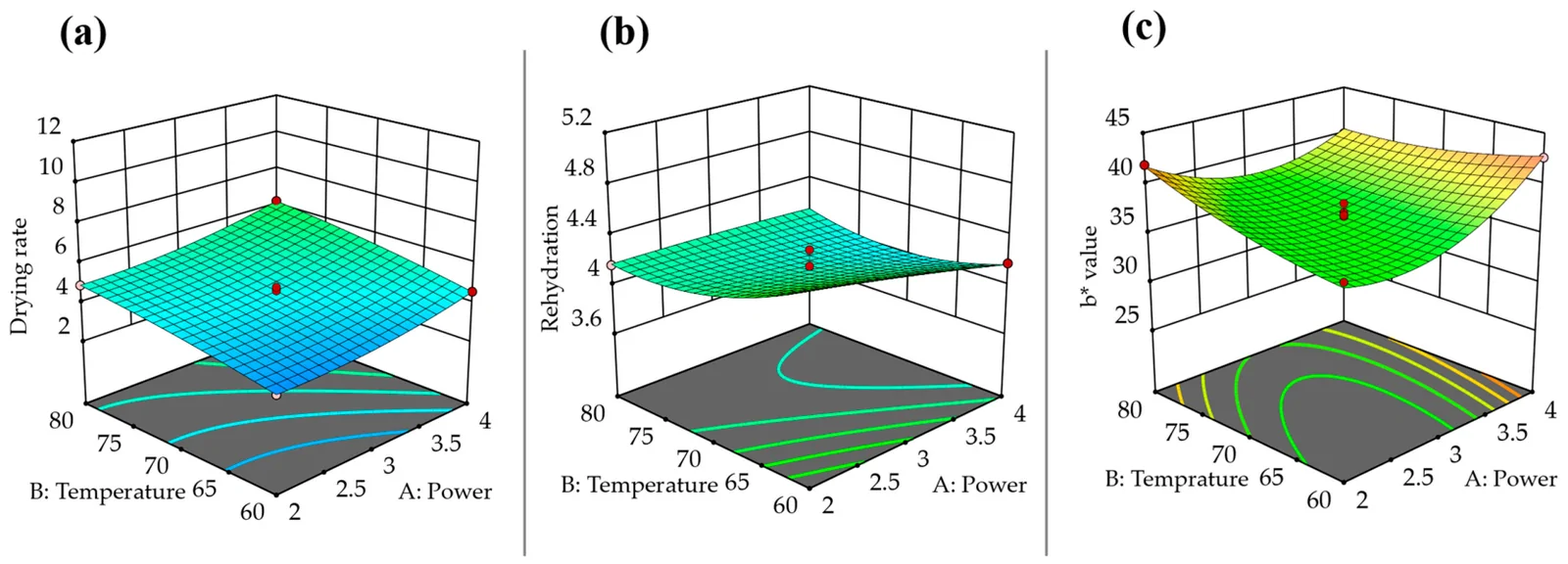
Three-dimensional response surface plots showing the effects of temperature and microwave power on drying rate (DR) in panel (**a**), rehydration capacity in panel (**b**), and b* value in panel (**c**) at a constant slice thickness. The red dots represent the experimental design data points, and the surface color gradient (from blue to red) indicates the magnitude of the response variables, ranging from minimum to maximum values.

Figure 2a shows the combined effects of microwave power (A) and drying temperature (B) on drying rate. Within the tested ranges (2–4 kW and 60–80 °C), drying rate generally increased as power and temperature rose. The response surface model fit the data well (R^2^ = 0.9970, *p* < 0.0001; F = 262.33). The lack-of-fit test was not significant (*p* = 0.0514; F = 6.48), and the coefficient of variation was low (CV = 3.69%). These results indicate that the model adequately describes the experimental data and provides reliable predictions for vacuum-microwave drying of Valencia orange slices.

Figure 2b shows that microwave power and temperature affected rehydration capacity nonlinearly. Capacity peaked at moderate conditions (approximately 70 °C and 3.0–3.5 kW) and decreased at higher intensities, possibly due to heat-related structural damage. The model showed a good fit (R^2^ = 0.9728, *p* = 0.0001), with a non-significant lack of fit (*p* = 0.7311) and a low coefficient of variation (CV = 2.21%). As shown in Figure 2c, b* values increased with processing intensity, indicating a shift toward more yellow-orange coloration. The color model also fit the data well (R^2^ = 0.9729, *p* = 0.0001), with a non-significant lack of fit (*p* = 0.7351) and a CV of 3.00%.

The developed models were used to maximize drying rate, rehydration capacity, and b* color value (Figure 2c). Based on the fitted quadratic models from the 17-run BBD experimental matrix (Table 1), multi-response numerical optimization was performed. The desirability analysis generated 49 numerical optimization solutions across the continuous factor space (detailed in Supplementary Table S5). Solution 1, exhibiting the highest overall desirability (D=0.718), was selected as the optimum operating condition: 4.0 kW microwave power, 80 °C drying temperature, and 2.2 mm slice thickness. Under these conditions, the models predicted a drying rate of 10.496 kg H_2_O/kg dry matter·h, a rehydration capacity of 4.749 g/g, and a b* value of 35.733. Three independent experiments were then conducted to validate the predictions. The measured values were 10.505 ± 0.420 kg H_2_O/kg dry matter·h for drying rate, 4.480 ± 0.173 g/g for rehydration capacity, and 35.82 ± 0.629 for b* value. These results were consistent with the model predictions, supporting the predictive performance of the response surface models (Table 2).

The mean results from three validation trials conducted under the optimal drying conditions (4.0 kW, 80 °C, and 2.2 mm) were compared with the model predictions. One-sample *t*-tests showed no significant differences between the measured and predicted values for drying rate, rehydration capacity, or b* value, supporting the predictive performance of the quadratic models under VMD conditions. The combination of high microwave power and temperature likely accelerated vapor generation and moisture diffusion, thereby reducing drying time while maintaining structural integrity and color. Similar effects of vacuum-microwave drying on porosity, rehydration, and pigment retention have been reported for citrus slices and other plant materials [18].

### 3.2. Optimization of Drying Parameters for Valencia Orange Slices in a Hot-Air Dryer Using the Response Surface Methodology (RSM)

The drying conditions for Valencia orange slices in a hot-air dryer were optimized using a Box–Behnken design. Air velocity, drying temperature, and slice thickness were the independent variables. Seventeen experimental runs evaluated drying rate, rehydration capacity, and a* color value (Table 3). All response models were significant (*p* < 0.0001), with non-significant lack of fit. The drying-rate model showed a strong fit (R^2^ = 0.9824, F = 43.41, CV = 9.36%, and lack-of-fit *p* = 0.4995). The rehydration-capacity model also fit the data well (R^2^ = 0.8415, F = 23.01, CV = 4.54%, and lack-of-fit *p* = 0.4227). The a* color model was significant, although its variability was higher (R^2^ = 0.8065, F = 18.06, CV = 22.14%, and lack-of-fit *p* = 0.9480). The response surface plots show how operating conditions affect drying rate and color during HAD. Factor coding followed standard normalization $X_{i}=\left(x_{i}-x_{0}\right)/\Delta x$ mapping factor levels to −1,0,+1. Model equations are presented in both coded and actual engineering units.

The regression models for the three main responses—drying rate, rehydration capacity, and a* color value—are presented in Equations (11)–(13). Drying rate was described by a quadratic model, while linear models were used for rehydration capacity and a* value. Together, these equations describe how the HAD process variables—air velocity (A), temperature (B), and slice thickness (C)—relate to each response and can be used to predict process outcomes:

DR = +1.20 + 0.0290 A + 0.3580 B − 0.7149C + +0.0053 AB − 0.0768 AC − 0.1243 BC − 0.1521 A^2^ + 0.0364 B^2^ + 0.3050 C^2^ (24)

Rehydration Capacity = +4.11 + 0.0020 A + 0.0831 B − 0.5413 C(25)

a* value = +6.99 − 0.2981 A+0.2190 B + 4.01 C(26)

A = Air Speed (m/s), B = Temperature (°C), and C = Slice Thickness (mm).

**Figure 3:**
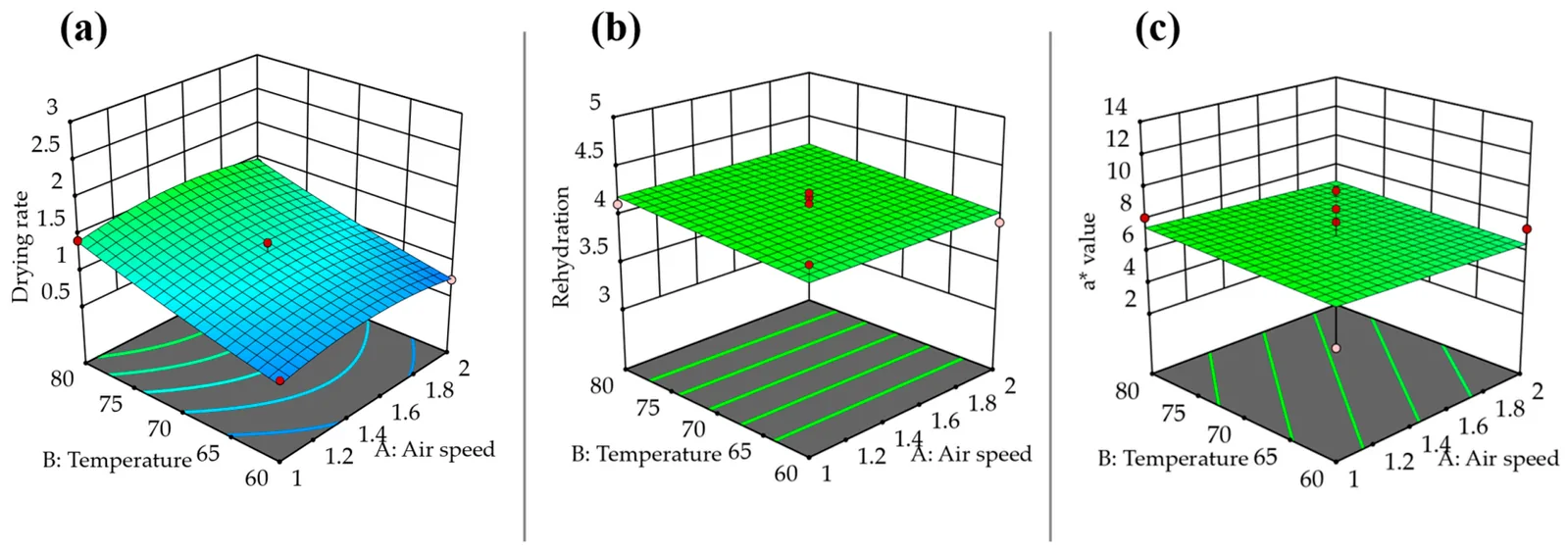
Three-dimensional response surface plots showing the effects of temperature and air speed on drying rate (DR) in panel (**a**), rehydration capacity in panel (**b**), and a* value in panel (**c**) at a constant slice thickness. The red dots represent the experimental design data points, and the surface color gradient (from minimum to maximum values) indicates the magnitude of the response variables, ranging from minimum to maximum values.

The response surface models described the effects of HAD conditions on drying rate, rehydration capacity, and color. Drying rate increased with temperature and air velocity, reaching its highest values at 80 °C and 2.0 m/s (Figure 3a). The quadratic model fit the data well (R^2^ = 0.9824, *p* < 0.0001; lack-of-fit *p* = 0.4995). Rehydration capacity increased with temperature and decreased with slice thickness (Figure 3b). Its linear model was significant (R^2^ = 0.8415, *p* < 0.0001; lack-of-fit *p* = 0.4227). The a* values ranged from approximately 6.0 to 12.0 and increased slightly with temperature (Figure 3c). The linear model was also significant (R^2^ = 0.8065, *p* < 0.0001; lack-of-fit *p* = 0.9480). The parity and regression plots (Figures S6–S8) showed close agreement between predicted and experimental values, supporting the models’ fit to the data. While the coefficients of determination for rehydration capacity ($R^{2}=0.8415$) and a* color value ($R^{2}=0.8065$) in HAD reflect moderate total variance explanation, both linear models were highly significant (p<0.0001) with non-significant lack-of-fit parameters (p>0.05). Such variance is typical in convective drying of multi-tissue citrus slices due to heterogeneous structural collapse and thermal pigment degradation. The predictive robustness was confirmed by physical validation trials, showing close agreement between experimental and model-predicted values (Table 4).

The experimental results agreed well with the fitted models, as shown by the ANOVA results. Multi-response optimization aimed to maximize drying rate and rehydration capacity while optimizing the a* color value using a desirability function. The optimal HAD conditions were 80 °C, an air velocity of 1.806 m/s, and a slice thickness of 2.00 mm. The 2.0 mm thickness was predicted by numerical optimization within the experimental range. In three validation trials, the measured values were close to the model predictions, confirming that RSM can be used to model and optimize HAD conditions (Table 4). Regarding the HAD process variables, the low linear coefficient for air velocity in Equation 24 demonstrates that its effect on drying rate was essentially plateaued across the tested range (1.5–2.0 m/s). The numerically predicted optimum air velocity of 1.806 m/s stems from minor statistical model variations rather than a substantial physical enhancement in mass transfer. In industrial operations, using a lower air speed within this range would be practically advantageous to reduce fan energy consumption without significantly compromising drying kinetics.

There was no significant difference between the experimental and predicted values for drying rate, rehydration capacity, or a* color value. One-sample *t*-tests confirmed close agreement for all three responses (Table S9a–c), supporting the regression models under the optimized HAD conditions. Unlike combined microwave–air drying, which can use lower air temperatures (~60 °C), pure convective drying requires greater thermal input. Accordingly, the optimum temperature in this study was 80 °C. Similar drying temperatures (70–80 °C) have been reported for kumquat slices and Washington navel orange slices [18,56]. The higher temperature likely helped moisture move through the orange tissue while maintaining acceptable rehydration and color. The experimental validation of the numerical optimization models demonstrated high predictive accuracy across all response variables (Table 2 and Table 4). One-sample *t*-tests confirmed no statistically significant differences between model-predicted and experimentally observed values (p>0.05). Furthermore, the absolute percentage errors remained exceptionally low for VMD (0.09% for drying rate, 5.66% for rehydration capacity, and 0.24% for b color value) and HAD (0.87% for drying rate, 2.24% for rehydration capacity, and 0.60% for a color value), verifying the operational robustness of the desirability optimization framework. For VMD rate, the adjusted $R^{2}$ (0.9931), predicted $R^{2}$ (0.9782), and adequate precision (48.62) confirmed a high signal-to-noise ratio despite borderline lack-of-fit (p=0.0514). In HAD, lower total variance explanation for rehydration ($R^{2}=0.8415$) and a* color ($R^{2}=0.8065,CV=22.14\%$) stems from heterogeneous tissue collapse and thermal degradation of citrus pigments under extended convective exposure. Desirability optimization was applied with equal weights (1.0). In the multi-response desirability optimization for HAD, minimizing the a* color coordinate (target ~3.0) was selected specifically to prevent excessive thermal browning and non-enzymatic Maillard reaction product accumulation, which inherently favored thinner slices due to shorter heat exposure. Nevertheless, it is recognized that while a* effectively tracks the degree of browning, the total color difference (ΔE) provides a more holistic assessment of overall visual color deviation relative to fresh tissue.

### 3.3. Some Quality Characteristics of Valencia Orange Slices Dried Using VMD and HAD Systems

**Figure 4:**
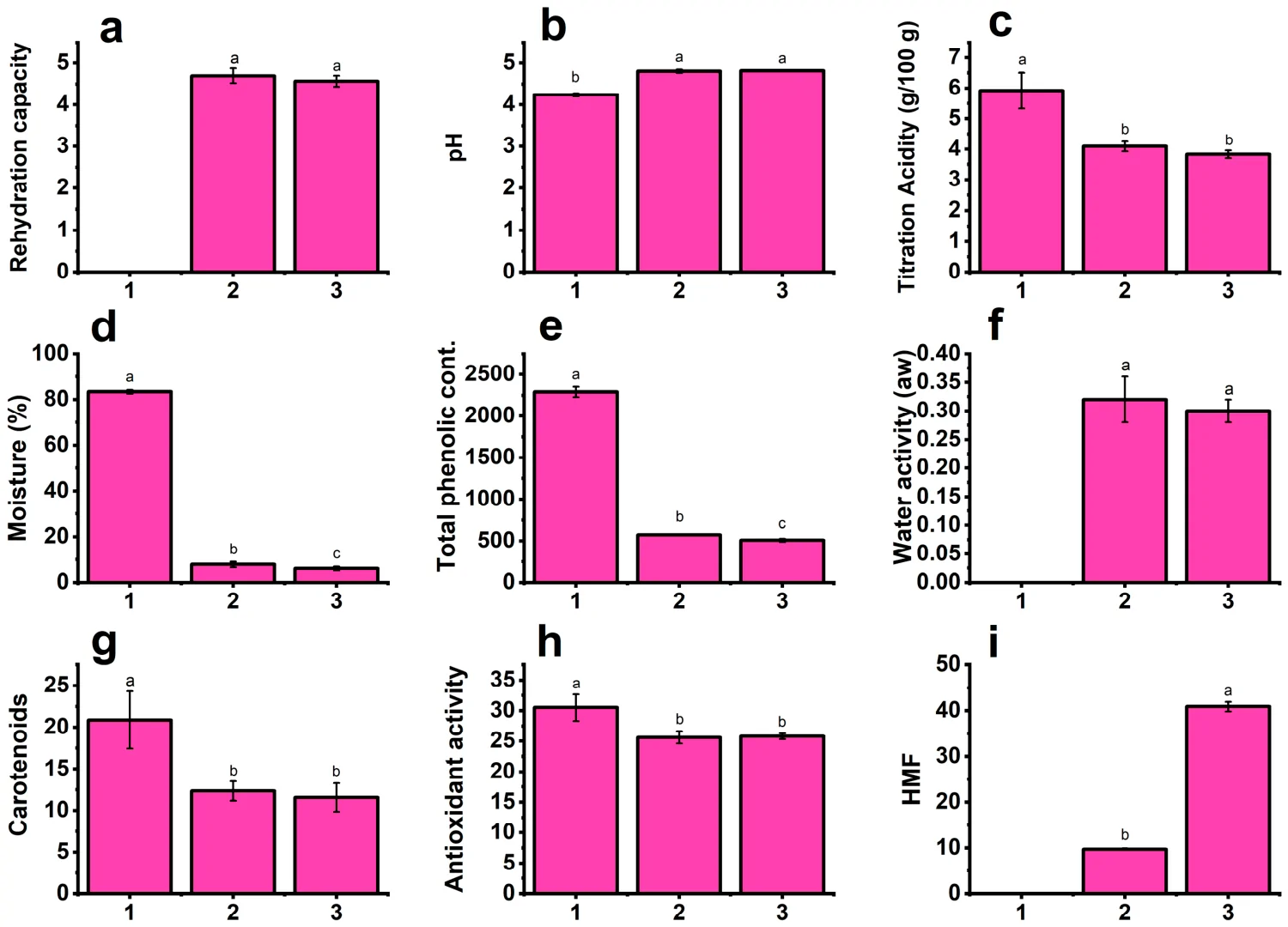
Quality parameters of fresh, vacuum-microwave-dried (VMD), and hot-air-dried (HAD) orange slices: (**a**) Rehydration capacity; (**b**) pH; (**c**) Titration acidity (g/100 g); (**d**) Moisture (%); (**e**) Total phenolic content; (**f**) Water activity (a_w_); (**g**) Carotenoids; (**h**) Antioxidant activity; (**i**) HMF. In each graph, the first, second, and third bars represent fresh, VMD-dried, and HAD-dried samples, respectively. Different letters above the bars indicate significant differences among treatments for each parameter (*p* < 0.05).

The effects of vacuum-microwave drying and hot-air drying on the physicochemical properties and bioactive compounds of Valencia orange slices were evaluated. The measured characteristics included rehydration capacity, pH, titratable acidity, moisture content, water activity, total phenolic and carotenoid contents, antioxidant activity, and hydroxymethylfurfural formation. The results are shown in Figure 4.

#### 3.3.1. Rehydration Capacity, Moisture Content (MC) and Water Activity (a_w_)

The rehydration capacity of Valencia orange slices did not differ significantly between VMD and HAD (*p* > 0.05, Figure 4a). Both methods produced similar rehydration ratios (≈4.6–4.7). Moisture content and water activity (a_w_) were significantly lower in dried slices than in fresh slices (*p* < 0.05, Figure 4d,f). HAD produced a slightly lower final moisture content (6.01 ± 0.24% DM) than VMD (8.04 ± 0.31% DM) (*p* < 0.05), while final water activity was measured as an evaluated quality attribute rather than a preset operational endpoint, remaining within the microbiologically stable range (a_w_ ≈ 0.30–0.32, *p* > 0.05) for both treatments. This additional 2.0% moisture reduction in HAD during the tail-end falling-rate period extended thermal exposure up to 220 min, thereby increasing specific energy consumption and accelerating thermal degradation markers (HMF and AGEs) without providing substantial additional storage stability compared to VMD. VMD also retained more total phenolics and produced less hydroxymethylfurfural (HMF; Figure 4i) than HAD. The porous structure formed during VMD may contribute to improved rehydration, as our previous study showed the porous structure of VMD-dried Washington navel orange slices shown by SEM microscopy [18]. Rehydration capacity showed no statistically significant difference between VMD (4.48±0.17 g/g) and HAD (4.63±0.07 g/g, p>0.05). All moisture content data are reported strictly on a dry weight basis (${g\;H}_{2}O/100\;g\;\mathrm{dry}\;\mathrm{matter}$).

#### 3.3.2. pH and Titratable Acidity

The pH of Valencia orange slices increased significantly during drying, while titratable acidity decreased (*p* < 0.05). Fresh slices had a pH of approximately 4.2, compared with approximately 4.8 in both VMD- and HAD-dried samples. The difference between the two drying methods was not significant (*p* > 0.05, Figure 4b). Titratable acidity decreased from approximately 5.9 g/100 g in fresh slices to 4.1 g/100 g after VMD and 3.8 g/100 g after HAD. This difference between drying methods was also not significant (*p* > 0.05). Overall, VMD and HAD had similar effects on pH and titratable acidity in Valencia orange slices. The difference between titratable acidity and HPLC citric acid reductions stems from the buffering capacity of organic acid salts present in the orange matrix.

#### 3.3.3. Total Phenolic, Carotenoid, and Antioxidant Activity

Drying significantly affected total phenolic content (TPC; *p* < 0.05, Figure 4e). Fresh samples had the highest TPC (≈2300 mg/100 g). TPC decreased after both treatments, but VMD samples retained significantly more phenolics (≈580 mg/100 g) than HAD samples (≈500 mg/100 g; *p* < 0.05). The shorter processing time and lower oxygen exposure during VMD may have reduced phenolic degradation [57]. Total carotenoid content also decreased significantly after drying, from ≈21.0 mg/100 g in fresh slices (Figure 4g; *p* < 0.05). The difference between VMD (≈12.5 mg/100 g) and HAD (≈11.8 mg/100 g) was not significant (*p* > 0.05; both marked “b”), suggesting that carotenoid loss was related mainly to heat exposure rather than drying method [58]. Antioxidant activity decreased from ≈30.5 in fresh slices to ≈25.8 after VMD and ≈25.9 after HAD (*p* < 0.05, Figure 4h). The difference between drying methods was not significant (*p* > 0.05), indicating similar antioxidant activity in both dried samples.

#### 3.3.4. Hydroxymethylfurfural (HMF) Formation

No HMF was detected in fresh Valencia orange slices. Drying led to a significant increase in HMF, with HAD samples (≈41.0 mg/kg) containing more than VMD samples (≈10.0 mg/kg; *p* < 0.05). This difference may reflect the longer exposure to heat and atmospheric oxygen during HAD, which can promote Maillard reactions and sugar degradation under acidic conditions [18]. Overall, VMD better limited HMF formation and retained more phenolics than HAD, while both methods produced similar rehydration capacity and acid retention. The lower HMF accumulation in VMD (10.0 mg/kg) compared to HAD (41.0 mg/kg) is possibly governed by the drastically reduced exposure duration (35 min vs. 200 min) and swift passage through intermediate water activity zones.

### 3.4. Phenolic Compounds Profile After Drying Processes

**Figure 5:**
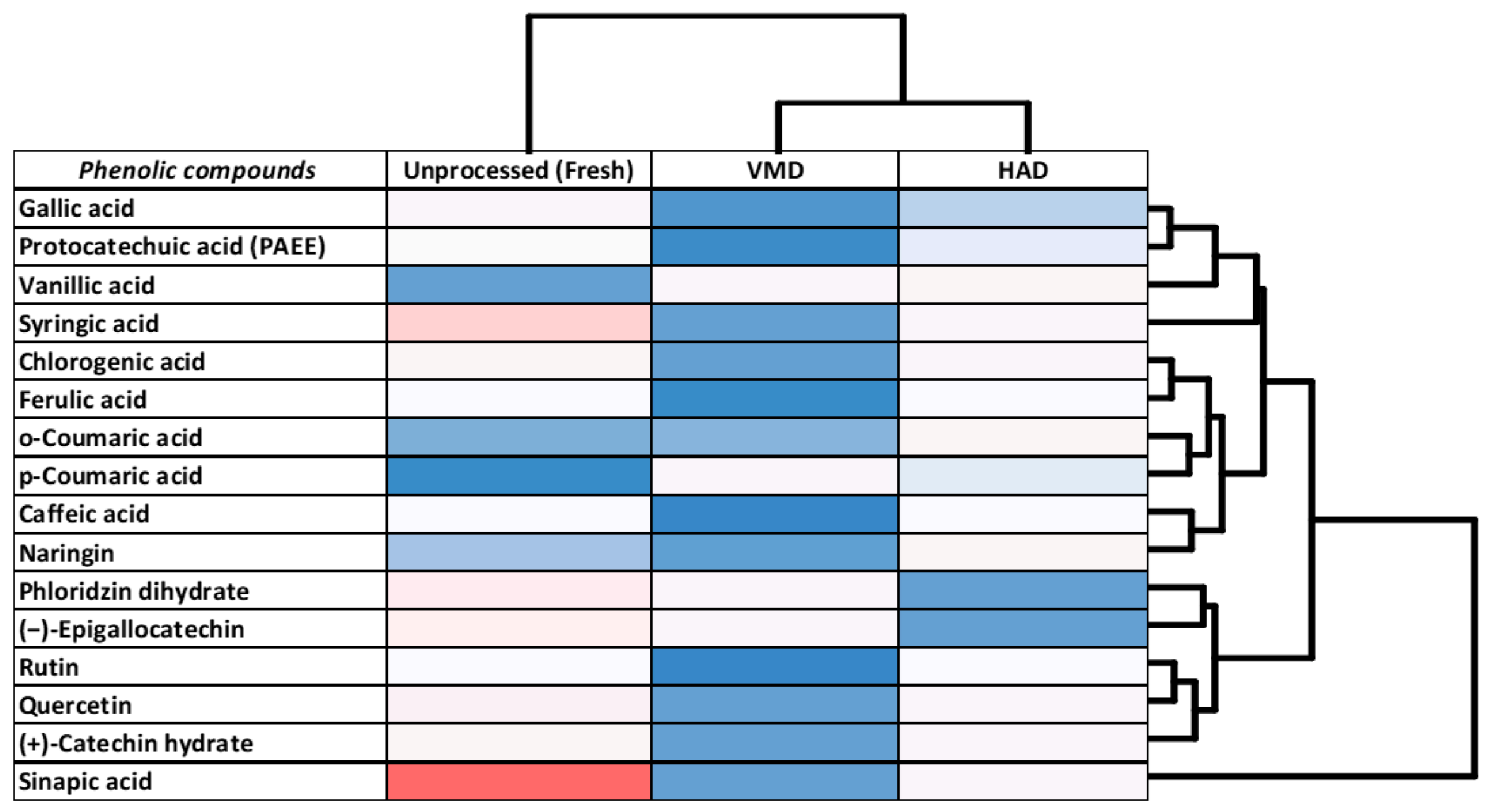
Hierarchical clustering and heat map of phenolic compounds in fresh, VMD, and HAD treated orange samples. The color gradient represents the relative abundance of phenolic compounds, where blue indicates higher concentrations, red indicates lower concentrations, and lighter shades represent intermediate or undetected values.

Phenolic compounds contribute substantially to the antioxidant capacity of plant-based foods. HPLC analysis identified several phenolic compounds in fresh and dried Valencia orange slices. Figure 5 presents a heatmap based on hierarchical clustering of standardized (z-score) values, showing differences in phenolic profiles among treatments. In fresh samples, the prominent compounds included the hydroxybenzoic acids protocatechuic acid (30.22 mg/100 g) and vanillic acid (19.56 mg/100 g), and the hydroxycinnamic acids o-coumaric acid (35.55 mg/100 g) and p-coumaric acid (17.47 mg/100 g). After drying, protocatechuic acid increased to 115.73 mg/100 g with vacuum-microwave drying and 48.11 mg/100 g with hot-air drying. Gallic acid also increased from 3.00 mg/100 g in fresh samples to 9.15 mg/100 g after VMD and 6.34 mg/100 g after HAD. These increases may be related to the breakdown of complex cinnamic derivatives and the release of bound phenolics during processing. By contrast, p-coumaric acid decreased from 17.47 mg/100 g in fresh samples to 2.23 mg/100 g after VMD and 5.92 mg/100 g after HAD. Chlorogenic acid (34.89 mg/100 g), caffeic acid (11.81 mg/100 g), and quercetin (19.79 mg/100 g), which were not detected in fresh samples, reached their highest concentrations after VMD. The flavan-3-ols also responded differently to drying. (−)-Epigallocatechin was not detected in fresh samples but reached 15.89 mg/100 g after VMD and 48.08 mg/100 g after HAD. Naringin was highest after VMD (17.77 mg/100 g), compared with 7.61 mg/100 g after HAD and 14.78 mg/100 g in fresh samples. Overall, the two drying methods produced different changes in the phenolic profile. VMD was associated with higher levels of a wider range of individual phenolic compounds than HAD. While VMD yielded higher retention across most phenolics, HAD retained significantly higher levels of (−)-epigallocatechin (48.08 mg/100 g) and p-coumaric acid (5.92 mg/100g).

### 3.5. Carotenoid Profile

**Figure 6:**
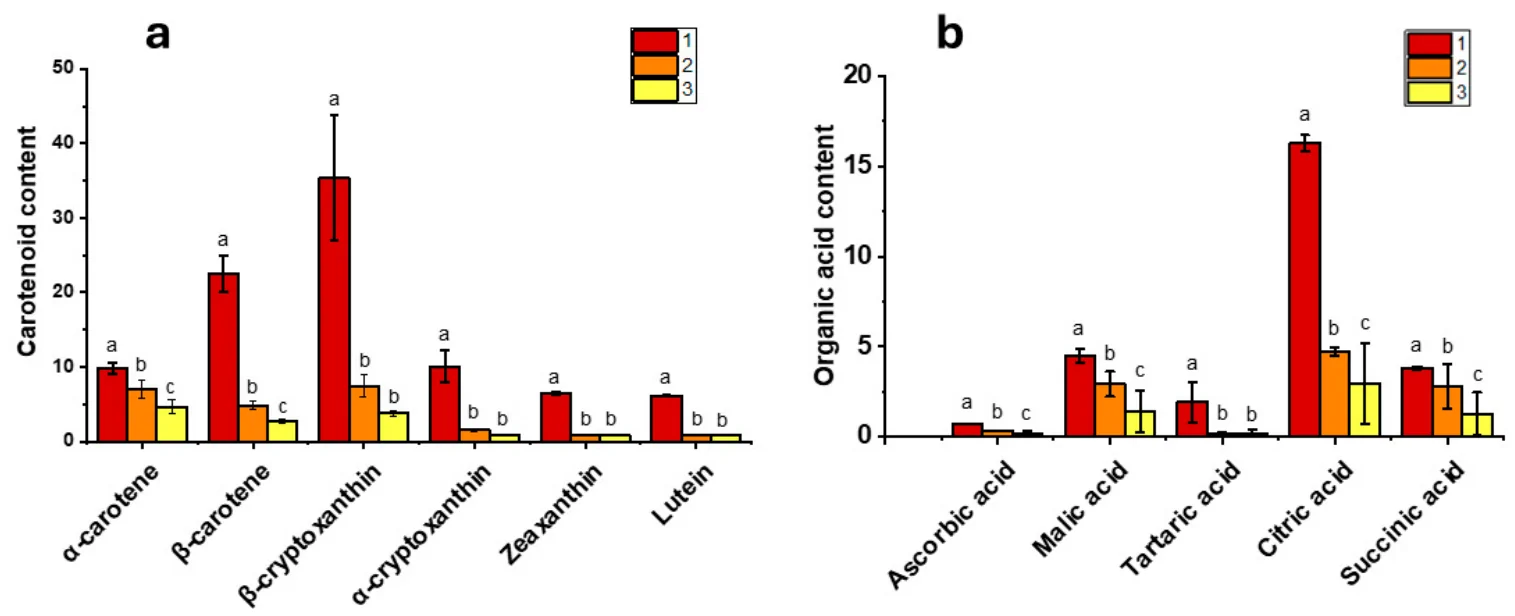
Carotenoid and organic acid profiles of orange slices: (**a**) Carotenoid content; (**b**) Organic acid content. Bars represent fresh, VMD-dried, and HAD-dried samples, respectively. Different letters indicate significant differences among treatments for each compound (*p* < 0.05).

Fresh Valencia orange slices contained several carotenoids, including β-cryptoxanthin, β-carotene, α-carotene, α-cryptoxanthin, zeaxanthin, and lutein (Figure 6a). β-Cryptoxanthin decreased from 35.39 ± 8.44 µg/g dry matter in fresh samples to 7.45 ± 1.45 µg/g after vacuum-microwave drying and 3.75 ± 0.34 µg/g after hot-air drying. These decreases were significant compared with fresh samples (*p* < 0.05), but the difference between drying methods was not significant (*p* > 0.05). α-Cryptoxanthin, zeaxanthin, and lutein also decreased significantly after drying, from 10.07 to 1.52 and 0.90 µg/g, 6.46 to 0.81 and 0.78 µg/g, and 6.17 to 0.86 and 0.84 µg/g, respectively (fresh, VMD, and HAD). For these compounds, differences between drying methods were not significant (*p* > 0.05). Overall, drying reduced carotenoid levels. VMD retained significantly more β-carotene (4.86 vs. 2.79 µg/g) and α-carotene (7.05 vs. 4.62 µg/g) than HAD (*p* < 0.05). HPLC analysis quantified six individual carotenoids, with VMD showing significantly higher retention of α-carotene (7.05 μg/g) and β-carotene (4.86 μg/g) compared to HAD (p<0.05).

### 3.6. Organic Acid Profile

The organic acid profile is shown in Figure 6b. Ascorbic acid (vitamin C) content in fresh Valencia oranges was 0.70 ± 0.021 g/100 g dry matter. After drying, it decreased to 0.32 ± 0.009 g/100 g dry matter with VMD and 0.20 ± 0.155 g/100 g dry matter with HAD (*p* < 0.05). Malic, tartaric, citric, and succinic acid contents also decreased significantly after drying. Malic acid decreased from 4.51 ± 0.395 g/100 g in fresh samples to 2.92 ± 0.697 g/100 g after VMD and 1.44 ± 1.160 g/100 g after HAD. Citric acid, the main organic acid in fresh samples (16.27 ± 0.449 g/100 g), decreased to 4.73 ± 0.224 g/100 g after VMD and 2.94 ± 2.247 g/100 g after HAD. Tartaric acid decreased from 1.94 ± 1.106 g/100 g to 0.16 ± 0.125 g/100 g after VMD and 0.19 ± 0.199 g/100 g after HAD; the difference between the drying methods was not significant (*p* > 0.05). Succinic acid decreased from 3.81 ± 0.082 g/100 g in fresh samples to 2.80 ± 1.205 g/100 g after VMD and 1.27 ± 1.196 g/100 g after HAD. Overall, the drying method significantly affected most organic acids (*p* < 0.05). VMD retained more ascorbic, malic, citric, and succinic acids than HAD (*p* < 0.05). These results show that drying conditions are important for preserving the organic acid profile and functional quality of orange products.

### 3.7. Sugar Content of Dried Orange Slices

In fresh Valencia orange samples, sucrose was the most abundant sugar (23.39 ± 1.31 g/100 g dry matter), accounting for approximately 42.6% of total sugars. Glucose and fructose accounted for 29.4% (16.14 ± 3.64 g/100 g dry matter) and 28.0% (15.38 ± 3.47 g/100 g dry matter), respectively (Table 5). Both vacuum-microwave drying and hot-air drying significantly reduced all three sugars compared with fresh samples (*p* ≤ 0.05). Sugar levels did not differ significantly between the drying methods (*p* > 0.05). Sorbitol was not presented in Table 5 as its concentration remained below the limit of quantification (<LOQ).

### 3.8. Color Value

Table 6 summarizes the color values of fresh and dried Valencia orange slices measured using the CIE Lab* system. Lightness (L*) increased significantly after drying, from 54.79 ± 1.042 in fresh samples to 83.20 ± 0.539 after VMD and 83.23 ± 1.238 after HAD (*p* < 0.05). The drying methods did not differ significantly in lightness (*p* > 0.05). Redness (a*) decreased significantly from 8.08 ± 0.451 in fresh samples to 1.76 ± 0.538 after VMD and 1.74 ± 0.392 after HAD (*p* < 0.05), with no significant difference between methods (*p* > 0.05). HAD resulted in greater total color difference (ΔE = 31.58 ± 0.943) and chroma difference (ΔC = 13.70 ± 0.627) than VMD (ΔE = 29.16 ± 0.536; ΔC = 6.59 ± 0.659; *p* < 0.05). Hue angle (h°) increased significantly after both drying treatments (*p* < 0.05) and was higher after VMD (87.78 ± 0.659) than after HAD (87.13 ± 0.718). Surface color was measured directly on intact slices, where the L* increase (54.79→83.20) reflects enhanced light scattering caused by microstructural pore formation during drying.

### 3.9. Effects of Drying Methods on Bioaccessibility of Bioactive Compounds and Intestinal Barrier Function

**Figure 7:**
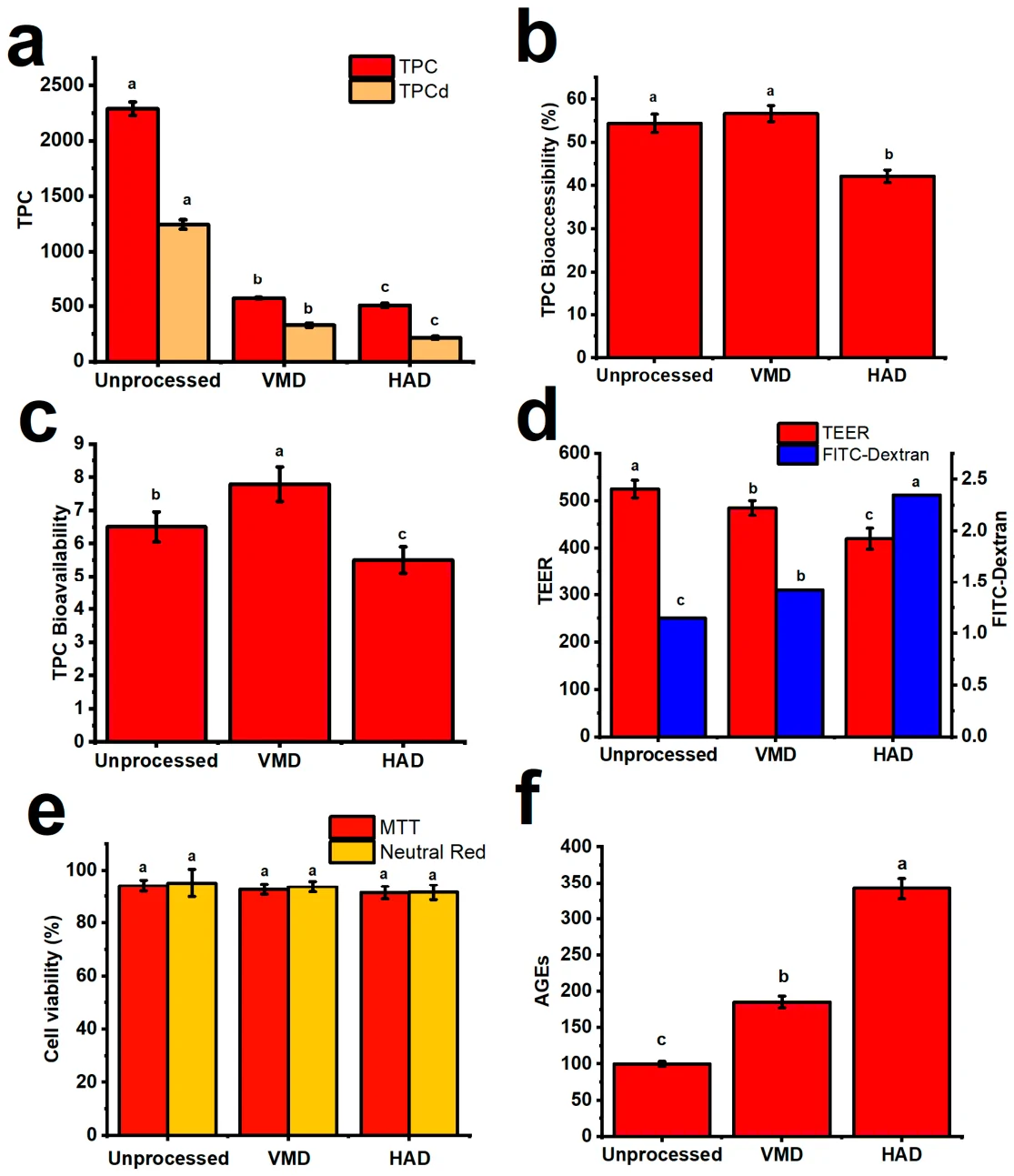
Effects of drying methods on bioactive compound bioaccessibility and Caco-2 intestinal barrier integrity after INFOGEST 2.0 in vitro digestion. (**a**) Total phenolic content before and after digestion in fresh (unprocessed), vacuum-microwave-dried (VMD; 4 kW, 80 °C, 2.228 mm), and hot-air-dried (HAD; 80 °C, 1.806 m/s, 2.00 mm) Valencia orange samples. (**b**) TPC bioaccessibility (%) after digestion. (**c**) TPC basolateral transepithelial transport efficiency (%) after digestion. (**d**) Transepithelial electrical resistance of Caco-2 monolayers exposed to digesta and FITC-Dextran permeability. (**e**) Cell viability measured by MTT assay; viability exceeded 90% in all treatments. (**f**) Total fluorescent advanced glycation end-products (AGEs; AU/g DM) across drying treatments. Data are presented as mean ± SD. Different letters indicate significant differences among treatments (*p* < 0.05).

Figure 7 shows the bioaccessibility of bioactive compounds in fresh (unprocessed), vacuum-microwave-dried (VMD; 4 kW, 80 °C, 2.228 mm), and hot-air-dried (HAD; 80 °C, 1.806 m/s, 2.00 mm) Valencia orange samples after INFOGEST 2.0 in vitro digestion, together with their effects on a Caco-2 intestinal barrier model. Before digestion, total phenolic content was highest in unprocessed samples. Drying reduced TPC, but VMD retained more phenolics after digestion than HAD. TPC bioaccessibility was approximately 56% for VMD, 54% for unprocessed samples, and 42% for HAD. The higher value for VMD may reflect greater phenolic release during digestion, while heat exposure during HAD may have contributed to phenolic degradation or reduced release. TPC transepithelial transport efficiency was also highest in VMD samples (7.8%), followed by unprocessed (6.5%) and HAD (5.2%) samples. MTT results showed cell viability above 90% in all groups, indicating that the digesta concentrations were not cytotoxic. TEER values were approximately 520 Ω·cm^2^ for unprocessed samples, 490 Ω·cm^2^ for VMD, and 420 Ω·cm^2^ for HAD. FITC-dextran permeability increased from approximately 1.0 in unprocessed samples to 1.4–1.5 in VMD and 2.1 in HAD samples. These results suggest that drying affected epithelial barrier integrity, with VMD maintaining it better than HAD. Finally, total fluorescent advanced glycation end-products (AGEs) like compound formation (Figure 7f) rose from ~100 AU/g DM in unprocessed samples to ~180 AU/g DM in VMD and peaked at ~340 AU/g DM in HAD. This sharp increase in HAD reflects a higher thermal load and Maillard reaction intensity, consistent with elevated HMF formation reported in Section 3.3.4 of this study. While VMD demonstrated higher overall phenolic bioaccessibility and reduced AGE-like compound accumulation, these comparative advantages should be interpreted as broad thermal intensity indicators, acknowledging the method-specific RSM optimization boundaries and minor differences in final dry-basis moisture content between treatments.

### 3.10. Drying

Kinetic Results of Valencia Orange Slices Dried Using VMD and HAD Systems

#### Drying Kinetics and Moisture Transfer Behavior

**Figure 8:**
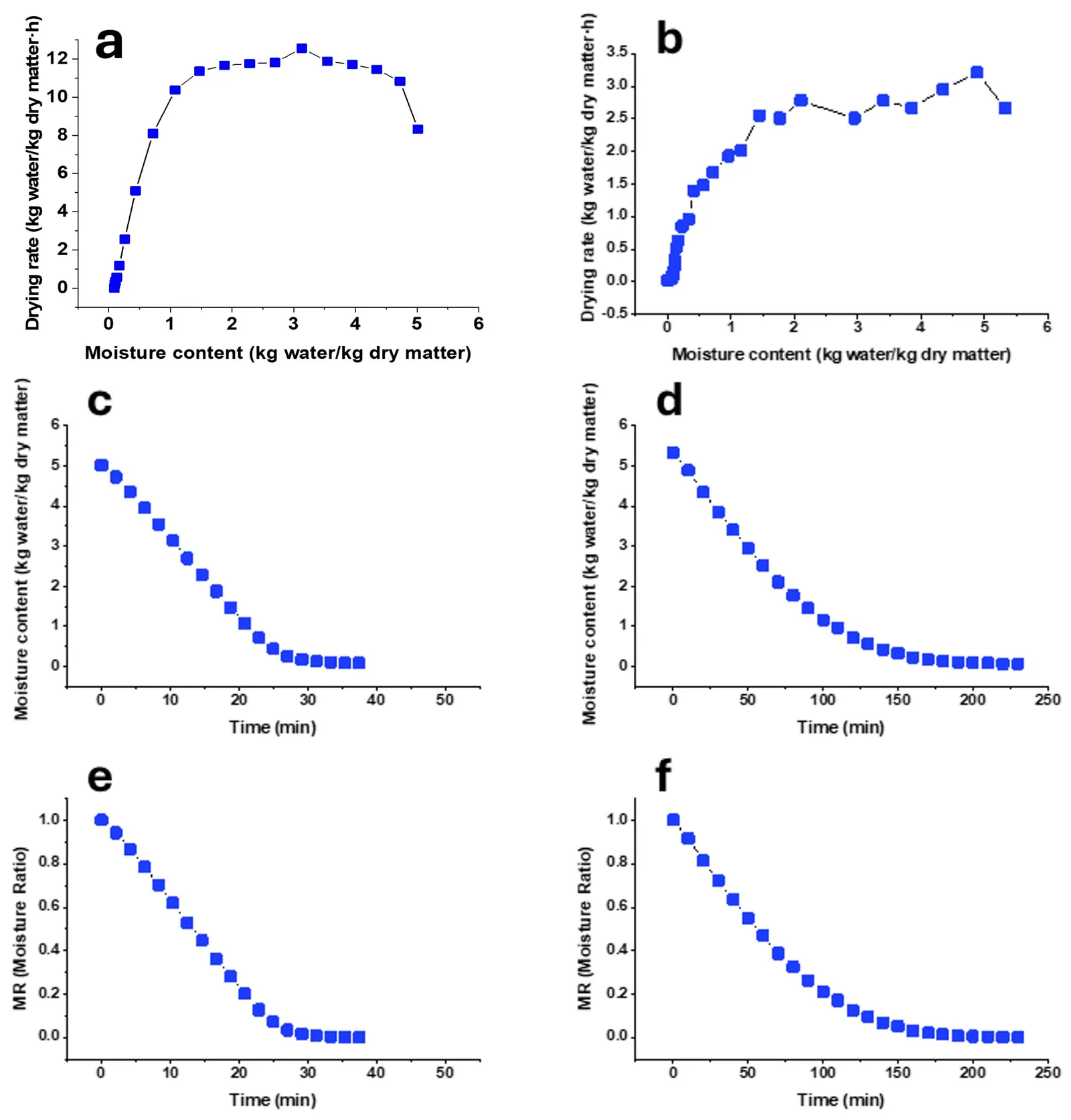
Variation in DR, MC, and MR of Valencia orange slices as a function of drying time under VMD and HAD conditions. Panels (**a**,**c**,**e**) represent VMD, whereas panels (**b**,**d**,**f**) represent HAD.

To guarantee direct comparability between the VMD and HAD systems, all drying trials were conducted using standardized initial batch masses (∼600 g), uniform slice thicknesses (∼2.0 mm), target final moisture levels (<10%), and identical water activity endpoints ($a_{w}<0.32$). The drying rate, moisture content, and moisture ratio profiles showed that the drying method influenced moisture transfer in Valencia orange slices. Under VMD, the DR increased rapidly at high MC, reaching $10.505\pm 0.420\;{\mathrm{kg}\;H}_{2}O/\mathrm{kg}\;\mathrm{DM}\cdot h$. Microwave energy heats the material volumetrically, generating internal vapor pressure that promotes moisture movement from the center to the surface. The DR then declined, indicating that drying occurred mainly in the falling-rate period. This pattern is typical of cellular food materials, in which internal diffusion is the main mechanism of moisture movement [59]. Under VMD, the MR fell rapidly to approximately 0.05 under 30 min, consistent with enhanced internal moisture transport and shorter drying times in microwave-vacuum systems. In contrast, HAD removed moisture more slowly. The DR increased gradually over a longer period, while the MR reached approximately 0.05 after about 200 min. Moisture content approached equilibrium after approximately 220 min. The longer drying time likely reflects the absence of volumetric heating and the lower heat-transfer rate in convective systems. Figure 8 shows the DR, MC, and MR of Valencia orange slices over time under VMD and HAD conditions.

### 3.11. Effective Moisture Diffusivity

D_eff values were determined as:

1.07 × 10^−9^ m^2^/s for VMD.
1.49 × 10^−10^ m^2^/s for HAD.

The difference was statistically significant (*p* < 0.05). The values fall within the typical range reported for food materials (10^−11^–10^−9^ m^2^/s) [60]. VMD produced an approximately sevenfold higher effective moisture diffusivity than HAD, likely because internal vapor-pressure gradients and structural expansion enhanced moisture transport. This result is consistent with studies reporting higher diffusivity during microwave-assisted than convective drying [61,62]. The VMD value (1.07 × 10^−9^ m^2^/s) was within the upper range reported for vacuum- and microwave-assisted drying of citrus, while the HAD value (1.49 × 10^−10^ m^2^/s) was consistent with slower moisture migration during conventional hot-air drying. Overall, the results indicate that VMD enhanced internal moisture transport relative to HAD.

The observed difference in effective moisture diffusivity between VMD ($1.07\times 10^{-9}{\;m}^{2}/s$) and HAD ($1.49\times 10^{-10}{\;m}^{2}/s$) can be fundamentally attributed to their distinct thermal energy transfer mechanisms and temperature dependence. In thin-layer drying of cellular plant matrices, moisture movement is thermally activated and follows an Arrhenius-type relationship ($D_{eff}=D_{0}\exp\left(-E_{a}/RT\right)$), where higher internal product temperatures decrease water viscosity and enhance molecular mobility. In conventional HAD, heat is transferred slowly from the surface inward, creating a thermal lag and requiring a higher activation energy ($E_{a}$) to drive paracellular moisture diffusion. In contrast, VMD combines volumetric microwave heating with reduced ambient pressure, generating rapid internal vaporization and localized vapor pressure buildup. This internal hydraulic pressure gradient significantly enhances mass transfer, effectively reducing the apparent activation energy threshold required for moisture migration through the orange peel and pulp tissues. Consequently, VMD achieves an upper-limit diffusivity value typical of high-intensity microwave-assisted processes without causing excessive surface thermal damage [4,12,60].

### 3.12. Mathematical Modeling of Drying Behavior

Among the ten thin-layer drying models evaluated, the Page model best described Valencia orange slices dried by VMD, with an R^2^ of 0.994, an RMSE of 0.0276, and a χ^2^ of 0.00085. The Diffusion model provided the best fit for HAD, with an R^2^ of 0.997, an RMSE of 0.01851, and a χ^2^ of 0.00039. The predominance of the falling-rate period indicates that internal diffusion was the main moisture-transfer mechanism [60]. The different best-fit models suggest that drying behavior varied with the method: rapid heating and vapor generation under VMD were described well by the Page model, whereas the Diffusion Approximation model best represented HAD. Statistical results for all ten models are presented in Table S2.

### 3.13. Energy, Exergy, Sustainability, and Environmental Assessment

Under the optimized vacuum-microwave drying conditions (microwave power, 4 kW; drying temperature, 80 °C; slice thickness, 2.228 mm), Valencia orange slices were dried for 35 min, yielding an average moisture removal of 0.5049 kg H_2_O per batch. Using the nominal energy approach, the energy input was calculated as 2.33 kWh batch^−1^ (8.40 MJ batch^−1^), corresponding to a mean experimental specific energy consumption of 4.12 kWh kg^−1^ H_2_O removed (ranging between 3.96 and 4.33 kWh kg^−1^ H_2_O, as detailed in Table S3) and a specific moisture extraction rate of 0.243 kg H_2_O kWh^−1^. Assuming an average product temperature of 40 °C and a dead-state temperature of 25 °C, the latent energy associated with water evaporation was estimated at 1.215 MJ batch^−1^, corresponding to an energy utilization efficiency of 16.22%. The associated evaporation exergy was 58.19 kJ batch^−1^, resulting in an evaporation-based exergy efficiency of 0.693%. The difference between nominal electrical energy input and evaporation exergy ($8.34{\;\mathrm{MJ}\;\mathrm{batch}}^{-1}$) reflects the total operational energy footprint comprising magnetron conversion losses, sensible heating of the fruit matrix and chamber walls, and condenser heat load [28]. Within this simplified operational framework, the resulting evaporation-based exergy efficiency ($\eta_{ex}=0.693\%$) serves solely as a localized phase-change indicator rather than a full-system exergy destruction analysis. The resulting simplified exergetic sustainability index was 1.007. At the optimized Valencia VMD condition, the operating chamber pressure was approximately 47.4 kPa absolute (474 mbar) at 80 °C, closely corresponding to the saturation pressure of water at this temperature. Accordingly, no separate contribution from non-condensable-gas pressure was estimated within the simplified assessment. For the environmental assessment, an electricity emission factor of 0.436 kg CO_2_-eq kWh^−1^ was applied to estimate electricity-related operational greenhouse-gas emissions of 1.02 kg CO_2_-eq batch^−1^, equivalent to 1.80 kg CO_2_-eq kg^−1^ H_2_O removed. These results constitute an operational assessment of energy consumption, evaporation-related exergy, simplified exergetic sustainability, and electricity-related greenhouse-gas emissions; they do not represent a full life-cycle carbon footprint or a comprehensive system-level exergy analysis. It should be emphasized that the resulting simplified exergetic sustainability index ($SI_{ex}=1.007$) is directly tied to the low evaporation-based exergy efficiency ($\eta_{ex}=0.693\%$). In thermal drying operations, when exergy analysis is restricted solely to moisture evaporation against nominal power input, $SI_{ex}$ values remain near unity, indicating high apparent exergy destruction/loss. A higher $SI_{ex}$ (>1.5) requires comprehensive system integration and heat recovery, which should be evaluated in future pilot-scale studies.

For comparison, under its optimized operating conditions (${80\;}^{\circ }C$, 1.806 m/s, 2.0 mm slice thickness), the HAD process demonstrated a lower mean specific energy consumption (SEC) of $2.32\pm 0.08{\;\mathrm{kWh}/\mathrm{kg}\;H}_{2}O$ (ranging from $2.24\;\mathrm{to}\;2.40{\;\mathrm{kWh}/\mathrm{kg}\;H}_{2}O$) and operational greenhouse-gas emissions of $1.01{\;\mathrm{kg}\;\mathrm{CO}}_{2}{\text{-}\mathrm{eq}/\mathrm{kg}\;H}_{2}O$ removed. The evaporation-based exergy efficiency for HAD was evaluated as 1.23%, corresponding to a simplified exergetic sustainability index (${\mathrm{SI}}_{\mathrm{ex}}$) of 1.012. Although HAD requires lower total electrical energy per unit mass of water evaporated due to simple resistance heating, it prolonged the thermal exposure time up to 220 min, compared to only 35 min in VMD.

From a techno-economic standpoint, a distinct trade-off exists between VMD and HAD processes. While HAD demonstrates lower nominal specific energy consumption values ($2.24–2.40{\;\mathrm{kWh}\;\mathrm{kg}}^{-1}H_{2}O$) due to simpler convective resistance heating, VMD exhibits higher energy intensity ($3.96–4.33{\;\mathrm{kWh}\;\mathrm{kg}}^{-1}H_{2}O$) driven by the concurrent electrical loads of magnetron generation, continuous vacuum pumping, and active moisture condensation. However, this elevated energy input in VMD yields an approximate 75–80% reduction in processing duration. In industrial applications, the higher electrical operational expenditure of VMD is offset by increased production throughput, reduced facility footprint, lower labor overhead, and superior retention of heat-sensitive bioactive compounds and visual attributes, justifying its adoption for high-value citrus products.

## 4. Discussion

**Figure 9:**
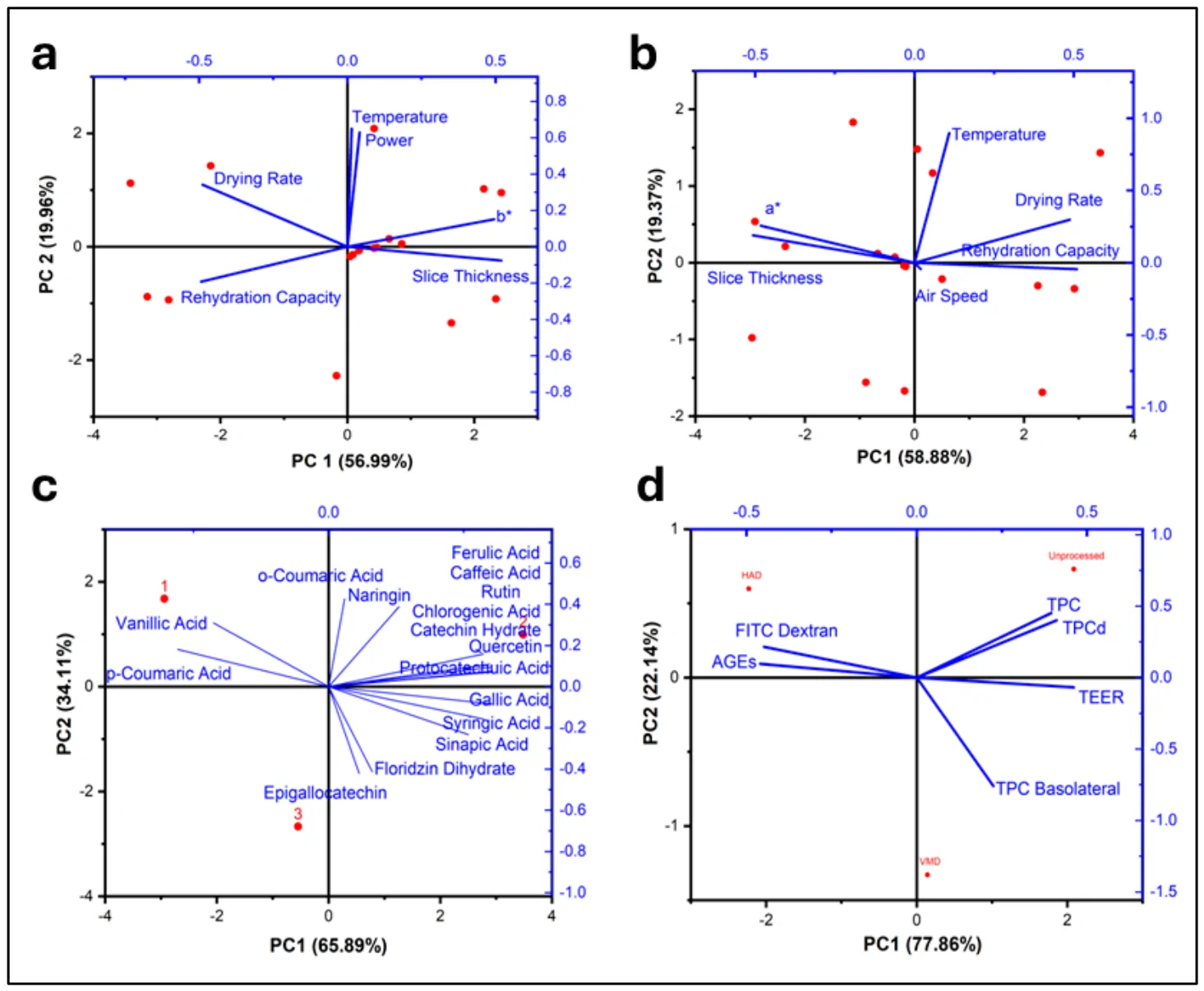
Principal component analysis (PCA) biplots showing process-response relationships and treatment discrimination across drying methods: (**a**) process variables and response attributes for vacuum-microwave drying (VMD); (**b**) process variables and response attributes for hot-air drying (HAD); (**c**) individual phenolic compound profile discrimination (1: Unprocessed/Fresh control, 2: VMD, 3: HAD); and (**d**) bioaccessibility, gastrointestinal digestion, Caco-2 epithelial barrier parameters, and thermal degradation markers (AGEs). The red dots in panels (**a**,**b**) represent the experimental design data points (observations).

This study showed that drying technology affected not only moisture removal but also the structure, chemical composition, bioactive-compound retention with bioaccessibility, and sustainability of Valencia orange slices. RSM models identified optimum conditions for both VMD and HAD. In VMD, higher microwave power and temperature increased the drying rate. Greater slice thickness reduced moisture removal. In HAD, slice thickness also reduced the drying rate. It was more directly influenced by temperature and air velocity. These differences reflect the distinct heat- and mass-transfer mechanisms. VMD promotes internal moisture movement through volumetric heating and vapor-pressure gradients. HAD transfers heat from the surrounding air before moisture diffuses through the product. The higher effective moisture diffusivity measured for VMD supports this distinction. Multivariate PCA biplots and Pearson matrices illustrated distinct separation patterns between drying treatments based on kinetics, bioactive retention, and thermal degradation markers. For VMD, the first two principal components explained 76.95% of the total variance (Figure 9a). Slice thickness was strongly negatively correlated with drying rate (r = −0.88; *p* < 0.001) and rehydration capacity (r = −0.82; *p* < 0.001). Drying rate was positively correlated with rehydration capacity (r = 0.75; *p* < 0.001). Drying rate was negatively correlated with the b* value (r = −0.73; *p* < 0.001), indicating that faster moisture removal was associated with changes in the yellow color coordinate. Microwave power and temperature had comparatively weak correlations with the measured responses. Slice thickness had the strongest linear association within the tested range. A similar pattern was observed for HAD. The first two principal components explained 78.25% of the total variance. Slice thickness was strongly negatively correlated with drying rate (r = −0.80; *p* < 0.001) and rehydration capacity (r = −0.91; *p* < 0.001). Drying rate and rehydration capacity were strongly positively correlated (r = 0.85; *p* < 0.001), while temperature and drying rate showed a moderate positive correlation (r = 0.47). These results indicate that slice thickness constrained drying in both systems. Temperature had a stronger relationship with drying rate in HAD. This is consistent with HAD’s dependence on heat transfer from the surrounding air. VMD heats the product volumetrically and promotes internal moisture movement. Overall, the multivariate results support the observed faster moisture removal and higher effective moisture diffusivity in VMD than in HAD.

The higher phenolic retention observed in VMD samples can be attributed to the reduced processing duration and oxygen-depleted environment, which together minimize polyphenol oxidase activity and thermal oxidation, as previously reported for microwave-vacuum processed citrus matrices [18,57]. Conversely, carotenoid degradation across both treatments was primarily driven by thermal isomerization and autoxidation under processing temperatures, aligning with earlier observations on heat-induced carotenoid breakdown in fruit drying [58].

For the phenolic compound profile (Figure 9c), the principal component analysis revealed that the first two principal components accounted for an exact cumulative variance of 100.00%, with PC1 explaining 65.89% and PC2 explaining 34.11% of the total variation. This complete two-dimensional spatial projection (PC1=65.89%, PC2=34.11%) reflects the mathematical property of projecting three treatment group means (N=3) in a two-dimensional space, capturing the dominant separation based on processing intensity and degradation patterns.

PCA biplots derived from group means (N=3) were utilized as qualitative multivariate visual mappings to illustrate treatment discrimination across drying kinetics, bioactive retention, and thermal markers, rather than inferential hypothesis testing yielding *p*-values. Protocatechuic acid, chlorogenic acid, caffeic acid, rutin, and quercetin clustered in the positive PC1 direction. Vanillic acid, p-coumaric acid, and epigallocatechin showed different loading patterns. These results were consistent with the HPLC analysis. The increased levels of protocatechuic acid, chlorogenic acid, caffeic acid, and quercetin may reflect the release or transformation of phenolics in bound or complex forms, alongside degradation. The decrease in p-coumaric acid further indicates that phenolic compounds differ in their thermal stability and transformation pathways. The transepithelial transport efficiency PCA (Figure 9d) provided an additional level of integrated assessment to the study by revealing the relationship between the chemical compositions of the dried samples and the responses observed after digestion and at the intestinal barrier. PC1 and PC2 explained 77.86% and 22.14% of the variance, respectively. TPC, post-digestion TPC, and basolateral TPC were positioned in the positive PC1 region; AGEs and FITC-dextran permeability were located in the opposite direction. TEER and basolateral TPC were associated with the VMD region, whereas the HAD sample was positioned towards the AGE and FITC-dextran vectors. This pattern aligns with the experimental results: higher TPC bioaccessibility and transepithelial transport efficiency were observed in VMD samples compared to HAD, while HAD samples exhibited lower TEER, higher FITC-dextran permeability, and significantly higher AGE formation. The relationship between variables related to transepithelial transport efficiency and intestinal barrier function is particularly significant, as it demonstrates that the preservation of chemical bioactives and the reduction in thermal reaction products occur simultaneously under VMD conditions. The approximately seven-fold increase in AGEs and the higher HMF concentration observed in HAD samples compared to the unprocessed sample indicate that a higher degree of thermal reaction occurs during conventional hot-air processing. In contrast, VMD significantly limited HMF formation and reduced AGE accumulation. These results are consistent with the shorter processing time and enhanced internal moisture transfer associated with VMD. These characteristics reduce the duration of exposure to conditions that promote sugar degradation and Maillard-type reactions.

The dominant negative correlation observed between slice thickness and both drying rate (r=−0.88) and rehydration capacity (r=−0.82) highlights a fundamental physical characteristic of dielectric heating under vacuum. In VMD, moisture removal is predominantly driven by internal volumetric vapor expansion and rapid hydraulic pressure gradients rather than slow convective surface heat transfer. Thinner slices (≈2.2 mm) offer significantly reduced resistance to mass transfer, facilitating rapid pressure-driven vapor discharge before microstructural collapse occurs. Therefore, it preserves matrix porosity and rehydration potential. Thicker slices impede internal vapor exit and create localized internal pressure buildup with uneven microwave attenuation. This non-uniform energy distribution often leads to localized overheating and structural crusting. It severely restricts subsequent water uptake during rehydration.

The strong negative correlation between drying rate and b* color value (r=−0.73) under VMD conditions highlights the biochemical sensitivity of citrus pigments to intensive electromagnetic energy input. This reduction in yellowness ($b^{*}$) is possibly governed by two concurrent phenomena. Thermal and oxidative degradation of dominant orange carotenoids (β-cryptoxanthin and violaxanthin) due to elevated localized power densities, alongside a mild onset of non-enzymatic browning reactions (Maillard precursor formation and thermal sugar degradation) occurring during the final low-moisture stage. Vacuum evacuation mitigates extensive atmospheric oxidation. Thus, localized thermal spikes inherent to rapid microwave absorption accelerate pigment thermal isomerization and localized caramelization. This leads to the observed subtle decrease in the $b^{*}$ coordinate.

The significantly higher accumulation of AGEs and HMF observed in HAD samples is directly governed by the interaction between surface temperature and localized water activity ($a_{w}$) dynamics during the falling-rate period. Conventional convective drying maintains a high surface temperature while holding the tissue within the critical intermediate $a_{w}$ reactivity window (0.55–0.80) for an extended duration. This prolonged residence in the highly mobile rubbery state—above the glass transition temperature ($T_{g}$)—accelerates Maillard precursor condensation and HMF formation. The rapid flash-evaporation inherent to VMD promotes fast volumetric cooling and swift moisture loss, causing bypassing of the optimal $a_{w}$ kinetic window for non-enzymatic browning and preserving the structural and chemical integrity of the citrus matrix. It should be noted that while VMD achieved target microbiological stability at ~8.0% DM moisture content (a_w_ = 0.32), HAD processing continued until reaching ~6.0% DM (a_w_ = 0.30). This difference in final moisture removal reflects the prolonged residence time required in convective drying to evaporate tightly bound capillary water, which intrinsically contributed to a higher cumulative thermal load, elevated HMF formation, and increased AGE accumulation in HAD samples.

**Figure 10:**
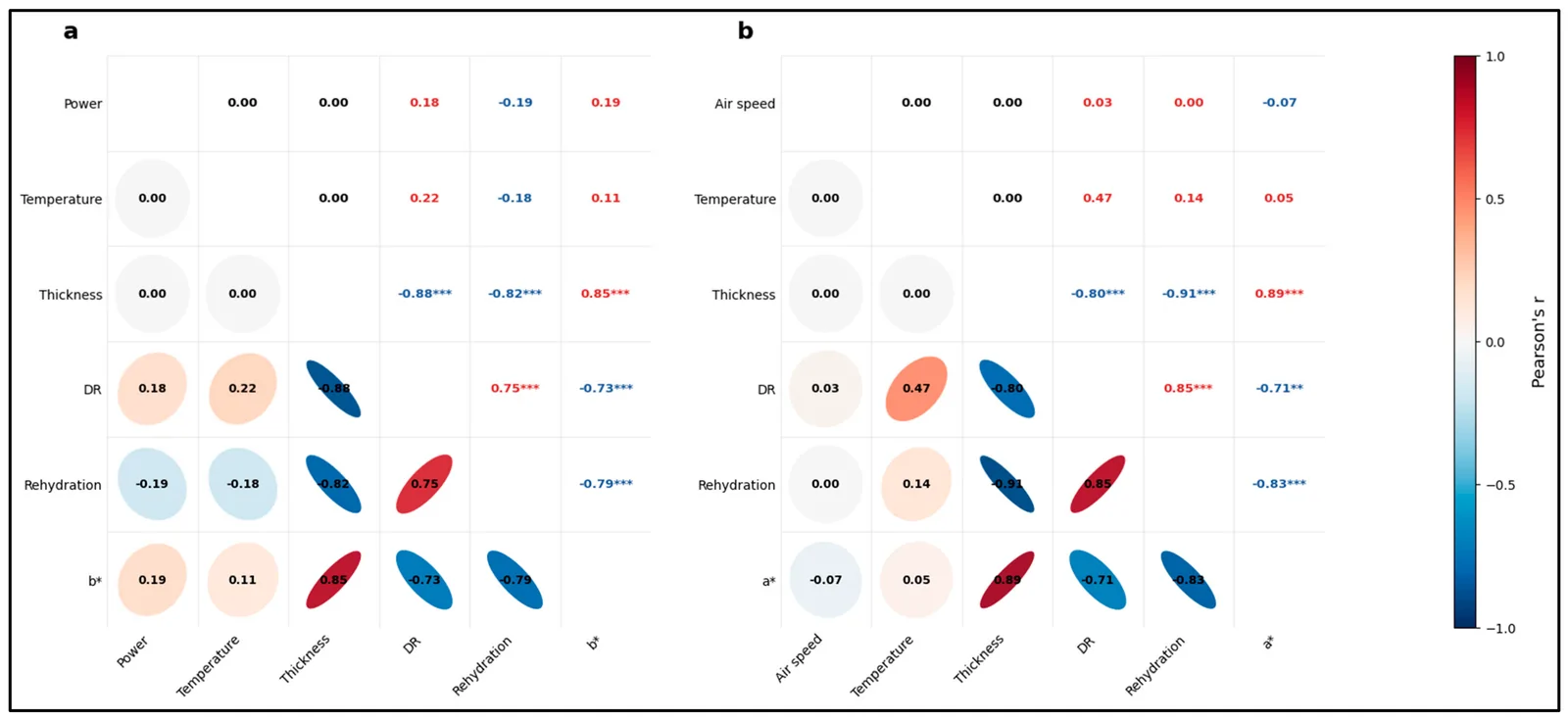
Pearson correlation matrices illustrating the linear relationships among process variables (power/air speed, temperature, and slice thickness) and drying/quality response attributes (drying rate [DR], rehydration capacity, b*, and a* color coordinates) for Valencia orange slices subjected to (**a**) VMD and (**b**) HAD. Note: Values within the matrix represent Pearson correlation coefficients (*r*). The color scale denotes the direction and strength of the correlation, ranging from blue (negative correlation) to red (positive correlation). Statistical significance levels are indicated by asterisks (** *p* < 0.01, and *** *p* < 0.001).

Overall, PCA and Pearson analysis (Figure 10) revealed that drying performance, product quality, phenolic composition, and gastrointestinal responses were not independent of one another. The results indicate that optimizing the drying process requires considering not only drying rate and energy aspects but also the preservation of bioactive compounds and the mitigation of thermally induced reactions. VMD technology simultaneously facilitated rapid internal moisture transfer, higher retention of various bioactive compounds, increased phenolic bioaccessibility, reduced formation of HMF and AGEs, and relatively better preservation of the intestinal barrier. In contrast, HAD required significantly longer moisture removal times and resulted in products with higher levels of thermal reaction products and increased intestinal permeability. Collectively, this study provides a mechanistic basis for evaluating drying technologies for Valencia orange slices—not merely through traditional drying criteria, but also by accounting for chemical and gastrointestinal outcomes.

From an industrial and techno-economic perspective, a distinct operational trade-off exists between VMD and HAD processing. Although HAD presents lower nominal specific energy consumption values due to conventional hot-air convective heating, VMD demands higher operational energy intensity ($3.96–4.33{\;\mathrm{kWh}/\mathrm{kg}\;H}_{2}O$) driven by concurrent magnetron emission, continuous vacuum pumping, and active condensation. However, this elevated energy input translates into an approximately 78% reduction in total processing time (35 min vs. 200–220 min). In industrial manufacturing, the higher electrical operational expenditure (OpEx) of VMD is offset by significantly enhanced production throughput, reduced equipment footprint, lower labor costs, and vastly superior retention of heat-sensitive bioactive phenolics, organic acids, and Caco-2 barrier integrity. Therefore, VMD stands out as a highly viable commercial strategy specifically for high-value functional citrus snacks and nutraceutical ingredients. Overall, this study provides a mechanistic basis for evaluating the drying of Valencia orange slices by considering not only traditional drying comparison criteria but also chemical, techno-economic, symptomatic, and gastrointestinal outcomes. It was observed that the results—including phenolic retention, HMF values, bioaccessibility, and drying kinetics—are generally consistent with the literature [4,6,18,63].

## 5. Conclusions

This study showed that vacuum-microwave drying is a promising alternative to hot-air drying for Valencia orange slices. Response surface methodology identified suitable conditions for both methods and showed that process variables influenced drying performance, rehydration, and color. VMD shortened drying time and enhanced moisture transfer. The Page model best described VMD, whereas the Diffusion Approximation model best described HAD. Drying altered the physicochemical properties and bioactive composition of the orange slices, but VMD generally preserved quality better than HAD. VMD-treated slices retained more total phenolics, several individual phenolic compounds, selected carotenoids, ascorbic acid, and organic acids. They also showed smaller color changes and less formation of heat-related compounds. Rehydration capacity and water activity were similar for both methods, but VMD provided a more favorable overall quality profile. After simulated gastrointestinal digestion, VMD samples showed greater phenolic bioaccessibility and transepithelial transport efficiency and better-maintained intestinal barrier integrity than HAD samples. HAD was associated with greater formation of HMF and fluorescent advanced glycation end-products, as well as greater changes in epithelial barrier properties. Overall, VMD demonstrated superior processing performance by achieving a 78% reduction in drying duration compared to HAD, while delivering a 2.4-fold increase in the bioaccessibility of key phenolic compounds, underscoring its efficacy for producing high-value dried citrus products. Future research should focus on sensory properties, consumer acceptability, pilot-scale techno-economic analysis, and life-cycle cost assessments (LCCA) to evaluate the industrial scalability and commercial viability of dried citrus products.

## Figures and Tables

**Table 1:** RSM-based drying performance analysis of Valencia orange slices in a vacuum-microwave dryer.

Sample ^(1)^	Factor 1	Factor 2	Factor 3	Response 1	Response 2	Response 3
	A: Power (kW)	B: Temperature (°C)	C: Slice Thickness (mm)	Drying Rate (DR) (kg H_2_O/kg·h)	Rehydration Capacity (g/g)	b* Value
VM1	3	70	4	4.518	4.071	34.564
VM2	2	60	4	3.435	4.587	37.128
VM3	2	70	2	8.941	4.922	30.397
VM4	3	60	6	2.464	4.306	41.314
VM5	4	70	6	4.351	3.884	43.676
VM6	3	60	2	8.210	4.666	28.729
VM7	4	80	4	6.217	4.072	39.986
VM8	3	70	4	4.784	4.153	36.898
VM9	3	70	4	4.611	4.099	38.083
VM10	3	70	4	4.533	3.997	37.166
VM11	2	70	6	3.015	3.771	42.673
VM12	4	60	4	4.559	4.176	42.601
VM13	2	80	4	4.883	4.161	41.878
VM14	3	70	4	4.544	4.285	36.759
VM15	3	80	6	3.285	3.687	42.210
VM16	3	80	2	10.496	5.031	31.385
VM17	4	70	2	10.009	4.516	35.569

^(1)^ VM = Sample code. Factor coding followed standard normalization $X_{i}=\left(x_{i}-x_{0}\right)/\Delta x$, mapping factor levels to −1,0,+1. Model equations are presented in both coded and actual engineering units.

**Table 2:** Comparison of experimental and predicted values obtained under optimal drying conditions in the VMD for Valencia oranges.

Responses	Experimental Data	Predicted Data
Drying Rate (DR)	10.505 ± 0.420	10.496
Rehydration Capacity	4.480 ± 0.173	4.749
b* color value	35.82 ± 0.629	35.733

**Table 3:** RSM-based drying performance analysis of Valencia orange slices in a hot-air dryer.

Sample ^(1)^	Factor 1	Factor 2	Factor 3	Response 1	Response 2	Response 3
	A: Air Speed (m/s)	B: Temperature (°C)	C: Slice Thickness (mm)	Drying Rate (DR) (kg H_2_O/kg Dry Matter.h)	Rehydration Capacity (g/g)	a* Value
TK1	2.0	70	6	0.685	3.548	10.156
TK2	1.5	60	2	1.808	4.710	2.787
TK3	1.5	80	2	2.818	4.788	3.880
TK 4	1.5	70	4	1.175	4.118	8.690
TK 5	2.0	60	4	0.734	3.927	7.438
TK 6	1.0	60	4	0.763	4.193	4.720
TK 7	1.5	70	4	1.076	4.068	9.810
TK 8	1.0	70	2	1.866	4.520	2.935
TK 9	1.0	70	6	0.704	3.400	12.342
TK 10	1.0	80	4	1.423	4.115	8.130
TK 11	1.5	70	4	1.093	4.234	7.860
TK 12	2.0	70	2	2.154	4.777	2.505
TK 13	1.5	70	4	1.321	4.185	4.348
TK 14	1.5	60	6	0.512	3.459	11.329
TK 15	2.0	80	4	1.415	3.993	5.643
TK 16	1.5	70	4	1.333	3.801	5.933
TK 17	1.5	80	6	1.333	4.057	10.372

^(1)^ TK = Sample code.

**Table 4:** Comparison of experimental and predicted values obtained under optimal drying conditions for Valencia oranges in a HAD.

Responses	Experimental Data	Predicted Data
Drying Rate (DR)	2.725 ± 0.003	2.749
Rehydration Capacity	4.631 ± 0.068	4.737
a* Color Value	3.000 ± 0.361	3.018

**Table 5:** Sugar content in fresh and dried Valencia orange slices.

Sugars	Valencia Orange Slices		
	Unprocessed (Fresh)	VMD	HAD
Glucose (g/100g dry matter)	16.14 ± 3.639 ^a^	13.04 ± 0.505 ^b^	12.70 ± 0.352 ^b^
Fructose (g/100g dry matter)	15.38 ± 3.468 ^a^	12.08 ± 0.838 ^b^	11.69 ± 0.240 ^b^
Sucrose (g/100g dry matter)	23.39 ± 1.307 ^a^	17.09 ± 0.388 ^b^	16.46 ± 1.041 ^b^

Values are expressed as mean ± standard deviation. Different superscript letters (^a, b^) within the same row indicate significant differences among drying treatments (*p* < 0.05).

**Table 6:** Color values of fresh and dried Valencia orange slices using different drying methods.

Color Values	Valencia Orange Slices		
	Untreated (Fresh)	VMD	HAD
L*	54.79 ± 1.042 ^b^	83.20 ± 0.539 ^a^	83.23 ± 1.238 ^a^
a*	8.08 ± 0.451 ^a^	1.76 ± 0.538 ^b^	1.74 ± 0.392 ^b^
b*	46.88 ± 0.528 ^a^	45.28 ± 0.885 ^b^	34.79 ± 0.862 ^c^
Delta e		29.16 ± 0.536 ^b^	31.58 ± 0.943 ^a^
Delta c		6.59 ± 0.659 ^b^	13.70 ± 0.627 ^a^
hue	80.21 ± 0.606 ^c^	87.78 ± 0.659 ^a^	87.13 ± 0.718 ^b^

The averages in the same row and indicated by different letters (a, b, and c) show statistically significant differences between values at a significance level of *p* < 0.05.

## Data Availability

The original contributions presented in this study are included in the article/Supplementary Material. Further inquiries can be directed to the corresponding author.

## Supplementary Materials

The following supporting information can be downloaded at: https://www.mdpi.com/article/10.3390/foods15214013/s1; Table S1: Commercial phenolic standards used for identification and external calibration in HPLC-PDA analysis; Table S2: Statistical comparison of ten thin-layer drying models used to determine the most appropriate mathematical description of moisture ratio changes during vacuum microwave drying and hot air drying of Valencia orange slices; Table S3: Raw data used for nominal specific energy consumption (SEC) calculations under optimum drying conditions for VMD and HAD systems; Table S4: RSM-Based ANOVA Analysis for Drying Valencia Oranges in a Vacuum Microwave Dryer; Table S5: Desirability-Based Numerical Optimization Solutions Generated by Design-Expert Software for Valencia Oranges in a Vacuum Microwave Dryer (Evaluated from the 17-Run BBD Experimental Model); Table S6a–c: T-test statistical outputs for drying rate, rehydration capacity, and b* values under optimal VMD conditions; Table S7: RSM-Based ANOVA Analysis of the Drying of Valencia Oranges in a Hot Air Dryer; Table S8: Desirability-Based Numerical Optimization Solutions Generated by Design-Expert Software for Valencia Oranges in a Hot Air Dryer (Evaluated from the 17-Run BBD Experimental Model); Table S9a–c: T-test statistical outputs for drying rate, rehydration capacity, and a* values under optimal HAD conditions; Figure S1: Residual diagnostic plots for the VMD drying rate model: (a) normal probability plot of residuals; (b) residuals versus predicted values; Figure S2: Residual diagnostic plots for the HAD drying rate model: (a) normal probability plot of residuals; (b) residuals versus predicted values; Figure S3: Comparison of experimental and RSM model-predicted values of drying rates in the vacuum microwave dryer for Valencia oranges, displaying 1:1 parity alignment within acceptable statistical prediction bands; Figure S4: Comparison of experimental and RSM model-predicted values of rehydration capacity in the vacuum microwave dryer for Valencia oranges, displaying 1:1 parity alignment within acceptable statistical prediction bands; Figure S5: Comparison of experimental and RSM model-predicted values for the b* color value in vacuum microwave drying of Valencia oranges, displaying 1:1 parity alignment within acceptable statistical prediction bands; Figure S6: Comparison of experimental and RSM model-predicted values of drying rates in the hot air dryer for Valencia oranges, displaying 1:1 parity alignment within acceptable statistical prediction bands; Figure S7: Comparison of experimental and RSM model-predicted values of rehydration capacity in the hot air dryer for Valencia oranges, displaying 1:1 parity alignment within acceptable statistical prediction bands; Figure S8: Comparison of experimental and RSM model-predicted values for the a* color value in the hot air dryer for Valencia oranges, displaying 1:1 parity alignment within acceptable statistical prediction bands.

## Abbreviations

AU	Arbitrary units
VMD	Vacuum-microwave drying
HAD	Hot-air drying
FITC-Dextran	Fluorescein isothiocyanate–dextran
TPC	Total phenolic content
RSM	Response surface methodology
HMF	Hydroxymethylfurfural
*D*_eff_	Effective moisture diffusivity
MR	Moisture ratio
DR	Drying rate
MC	Moisture content
R^2^	Coefficient of determination
RMSE	Root mean square error
SEM	Standard error of mean
χ^2^	Reduced chi-square
CV	Coefficient of variation
ANOVA	Analysis of variance
PDA	Photodiode array detector
HPLC	High-performance liquid chromatography
RID	Refractive index detector
DPPH	2,2-Diphenyl-1-picrylhydrazyl
GAE	Gallic acid equivalent
TE	Trolox equivalent
BHT	Butylated hydroxytoluene
AOAC	Association of Official Analytical Chemists
CIELAB	Commission Internationale de l’Éclairage Lab* Color System
ΔE	Total color difference
ΔC	Chroma difference
aw	Water activity
NaOH	Sodium hydroxide
H_2_SO_4_	Sulfuric acid
SPSS	Statistical package for the social sciences
