# Supplementary material for: Vacuum-Microwave vs. Hot-Air Drying of Valencia Orange Slices: Evaluation of Process Optimization and Drying Kinetics with Bioactive Bioaccessibility, Intestinal Barrier Function, Advanced Glycation End-Products, and Sustainability
Source: Foods. 2026 Nov 9;15(21):4013. doi: 10.3390/foods15214013 (PMC13762541; doi:10.3390/foods15214013)
Supplement: Supplementary file 1 [file foods-15-04013-s001.zip › foods-4628855-supplementary.pdf]

## Supplementary

**Table S1.** Commercial phenolic standards used for identification and external calibration in HPLC-PDA analysis

| | Phenolic compound name |
|---|---|
| **1** | Gallic acid |
| **2** | Chlorogenic acid |
| **3** | (+) Catechin hydrate |
| **4** | (-) Epigallocatechin |
| **5** | (-) Epicatechin |
| **6** | Vanillic acid |
| **7** | Syringic acid |
| **8** | Caffeic acid |
| **9** | Sinapic acid |
| **10** | Isoquercetin |
| **11** | Ferulic acid |
| **12** | PAEE (3,4-dihidroksibenzoic acid, protocatechuic acid) |
| **13** | Quercetin |
| **14** | Neohesperidin |
| **15** | Rutin |
| **16** | p-coumaric acid |
| **17** | phloridzin dihydrate |
| **18** | Hesperidin |
| **19** | Luteolin |
| **20** | Naringin |
| **21** | o-coumaric acid |
| **22** | Myricetin |
| **23** | Naringenin |
| **24** | Kaempferol |

**Table S2.** Statistical comparison of ten thin-layer drying models used to determine the most appropriate mathematical description of moisture ratio changes during vacuum microwave drying and hot air drying of Valencia orange slices.

| Model | Constants and Coefficients | Hot Air Drying | Vacuum Microwave Drying |
|---|---|---|---|
| Lewis (Newton) MR=exp(-k*t) | *k* | 0.021 | 0.068 |
| | $X^2$ | 0.00303 | 0.00967 |
| | *RMSE* | 0.05383 | 0.09573 |
| | $R^2$ | 0.972 | 0.924 |
| Page MR=exp(-k*t^n) | *k* | 0.004 | 0.008 |
| | $X^2$ | 0.00091 | 0.00085 |
| | *RMSE* | 0.0288 | 0.0276 |
| | $R^2$ | 0.998 | 0.994 |
| Henderson and Pabis | *k* | 0.023 | 0.076 |
| | *a* | 1.09 | 1.119 |
| | $X^2$ | 0.00227 | 0.00766 |

| | | | |
|---|---|---|---|
| MR=a*exp(-k*t) | *RMSE* | 0.04551 | 0.08279 |
| | $R^2$ | 0.980 | 0.944 |
| Modified Henderson and Pabis<br>MR=a*exp(-k*t)+b*exp(-g*t)+c*exp(-h*t) | *k* | 0.023 | 0.076 |
| | *a* | 0.304 | 0.378 |
| | *g* | 0.023 | 0.076 |
| | *b* | 0.487 | 0.391 |
| | *h* | 0.023 | 0.076 |
| | *c* | 0.300 | 0.370 |
| | $X^2$ | 0.00280 | 0.00994 |
| | *RMSE* | 0.04552 | 0.08245 |
| | $R^2$ | 0.980 | 0.944 |
| Logarithmic<br>MR=a*exp(-k*t)+c | *k* | 0.017 | 0.039 |
| | *a* | 1.172 | 1.487 |
| | *c* | -0.122 | -0.417 |
| | $X^2$ | 0.00080 | 0.00236 |
| | *RMSE* | 0.02630 | 0.04457 |
| | $R^2$ | 0.994 | 0.984 |
| Two term<br>MR=a*exp(-k*t)+b*exp(-c*t) | *k* | 0.023 | 0.076 |
| | *a* | 0.55 | 0.571 |
| | *c* | 0.023 | 0.076 |
| | *b* | 0.540 | 0.567 |
| | $X^2$ | 0.0025075 | 0.008609 |
| | *RMSE* | 0.04551278 | 0.08244 |
| | $R^2$ | 0.980 | 0.944 |
| Two-term Exponential<br>MR=a*exp(-k*t)+(1-a)*exp(-k*a*t) | *k* | 49.300 | 0.110 |
| | *a* | 0.000432 | 2.104 |
| | $X^2$ | 0.00316 | 0.00242 |
| | *RMSE* | 0.05374 | 0.04651 |
| | $R^2$ | 0.972 | 0.982 |
| Diffusion Approximation<br>MR=a*exp(-k*t)+(1-a)*exp(-k*b*t) | *k* | 0.041 | 0.149 |
| | *a* | -51.542 | -93.101 |
| | *b* | 0.984 | 0.988 |
| | $X^2$ | 0.00039 | 0.00205 |
| | *RMSE* | 0.01851 | 0.04156 |
| | $R^2$ | 0.997 | 0.986 |
| Verma<br>MR=a*exp(-k*t)+(1-a)*exp(-g*t) | *k* | 0.04 | 0.149 |
| | *a* | -143.229 | -178 |
| | *g* | 0.04 | 0.148 |
| | $X^2$ | 0.03097 | 0.00239 |
| | *RMSE* | 0.16412 | 0.04483 |
| | $R^2$ | 0.997 | 0.986 |
| Wang and Singh<br>MR=1+a*t+b*t^2 | *a* | -0.015 | -0.047 |
| | $b \times 10^{-5}$ | 5.70 | 51.00 |
| | $X^2$ | 0.36317 | 0.07071 |
| | *RMSE* | 0.57584 | 0.25154 |
| | $R^2$ | 0,999 | 0.984 |

**Table S3.** Raw data used for nominal specific energy consumption (SEC) calculations under optimum drying conditions for VMD and HAD systems.

| Drying method | Replicate | Fresh weight (g) | Dry weight (g) | Drying time (h) | Removed water (kg) | Specific Energy Consumption (kWh/kg removed water) |
|---|---|---|---|---|---|---|
| VMD | 1 | 614.32 | 110.91 | 0.5139 | 0.5034 | 4.08 |
| VMD | 2 | 620.99 | 110.12 | 0.5056 | 0.5109 | 3.96 |
| VMD | 3 | 613.66 | 113.23 | 0.5417 | 0.5004 | 4.33 |
| HAD | 1 | 544.20 | 98.41 | 2.0036 | 0.4458 | 2.40 |
| HAD | 2 | 557.03 | 101.31 | 2.0042 | 0.4557 | 2.35 |
| HAD | 3 | 583.75 | 104.09 | 2.0078 | 0.4797 | 2.24 |

Note: Nominal power values were 4.0 kW for VMD and 0.535 kW for HAD.

**Table S4.** RSM-Based ANOVA Analysis for Drying Valencia Oranges in a Vacuum Microwave Dryer

| Source | Sum of Squares | df | Mean Square Error | F-value | p-value |
|---|---|---|---|---|---|
| **Drying Rate** | | | | | |
| Model | 95.95 | 9 | 10.66 | 262.33 | < 0.0001 |
| Residual | 0.2845 | 7 | 0.0406 | | |
| Lack of Fit | 0.2359 | 3 | 0.0786 | 6.48 | 0.0514 |
| Pure Error | 0.0486 | 4 | 0.0121 | | |
| Total | 96.23 | 16 | | | |
| R2 = 0.9970 | | | | | |
| CV%=3.69 | | | | | |
| **Rehydration Capacity** | | | | | |
| Model | 2.22 | 9 | 0.2461 | 27.86 | 0.0001 |
| Residual | 0.0618 | 7 | 0.0088 | | |
| Lack of Fit | 0.0156 | 3 | 0.0052 | 0.4499 | 0.7311 |
| Pure Error | 0.0462 | 4 | 0.0116 | | |
| Total | 2.28 | 16 | | | |
| R2 = 0.9728 | | | | | |
| CV%=2.21 | | | | | |

| | | | | | |
|---|---|---|---|---|---|
| **b* value** | | | | | |
| Model | 321.93 | 9 | 35.77 | 27.91 | 0.0001 |
| Residual | 8.97 | 7 | 1.28 | | |
| Lack of Fit | 2.24 | 3 | 0.7462 | 0.4433 | 0.7351 |
| Pure Error | 6.73 | 4 | 1.68 | | |
| Total | 330.90 | 16 | | | |
| R2 = 0.9729 | | | | | |
| CV%=3.00 | | | | | |

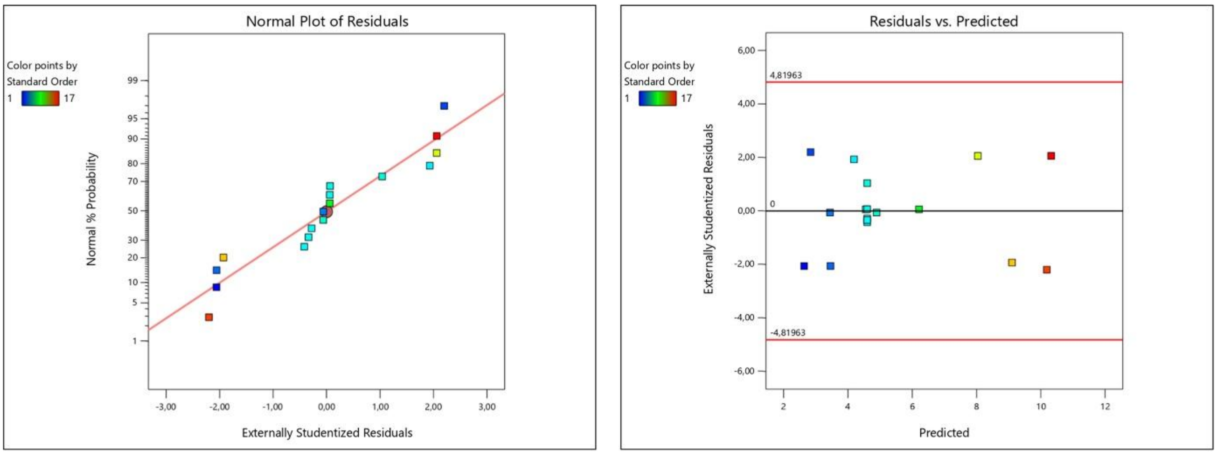


a) b)

**Figure S1.**Residual diagnostic plots for the VMD drying rate model: (a) normal probability plot of residuals; (b) residuals versus predicted values.

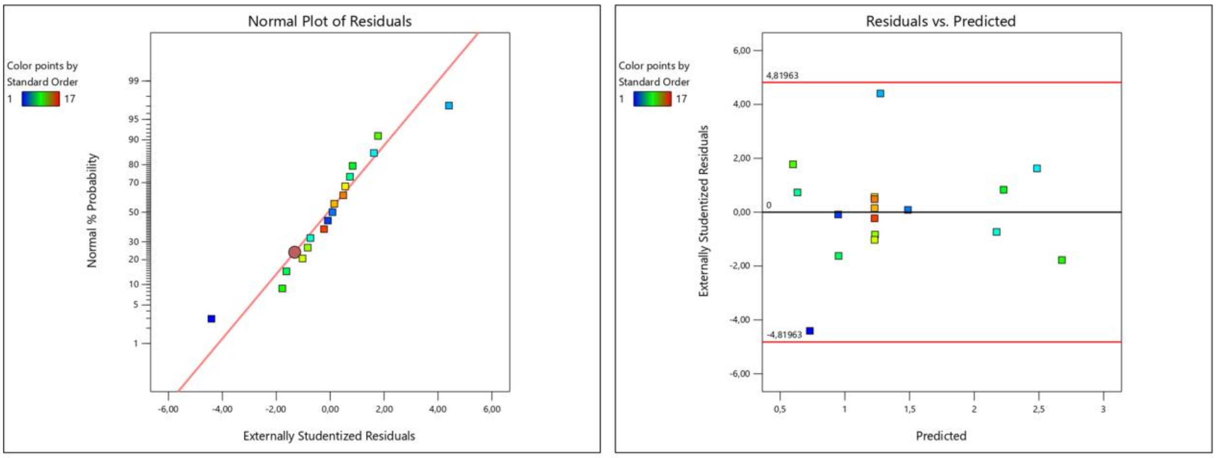


a) b)

**Figure S2.** Residual diagnostic plots for the HAD drying rate model: (a) normal probability plot of residuals; (b) residuals versus predicted values.

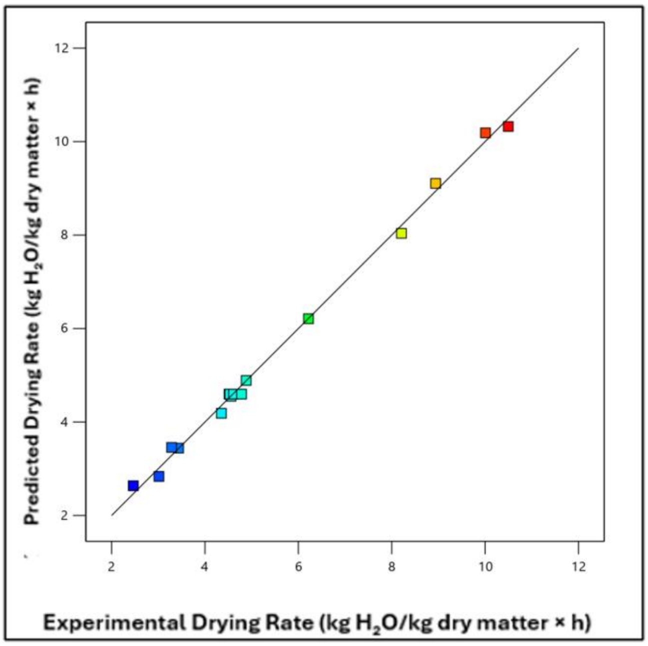


**Figure S3.** Comparison of experimental and RSM model-predicted values of drying rates in the vacuum microwave dryer for Valencia oranges, displaying 1:1 parity alignment within acceptable statistical prediction bands.

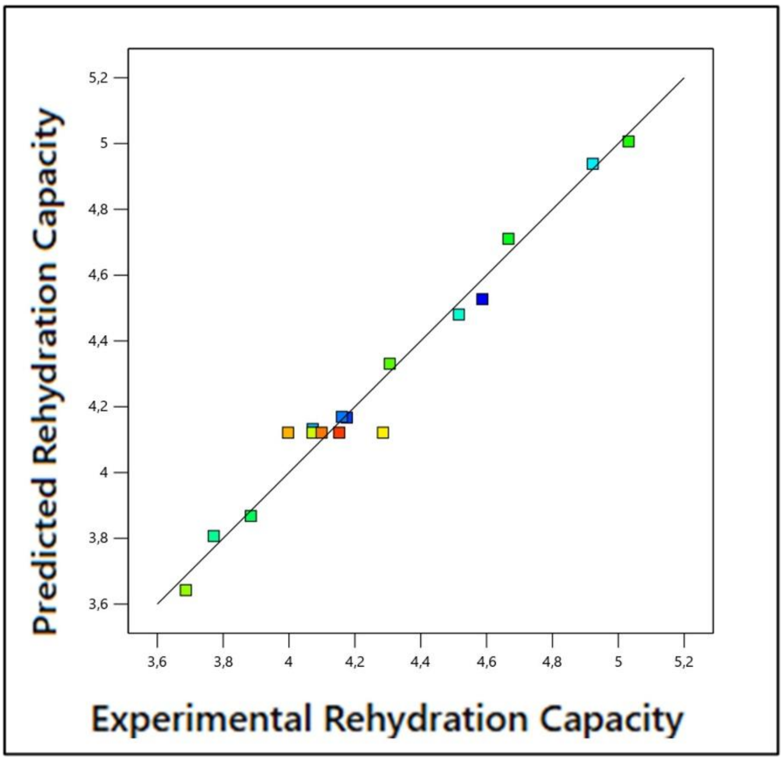


**Figure S4.** Comparison of experimental and RSM model-predicted values of rehydration capacity in the vacuum microwave dryer for Valencia oranges, displaying 1: 1 parity alignment within acceptable statistical prediction bands.

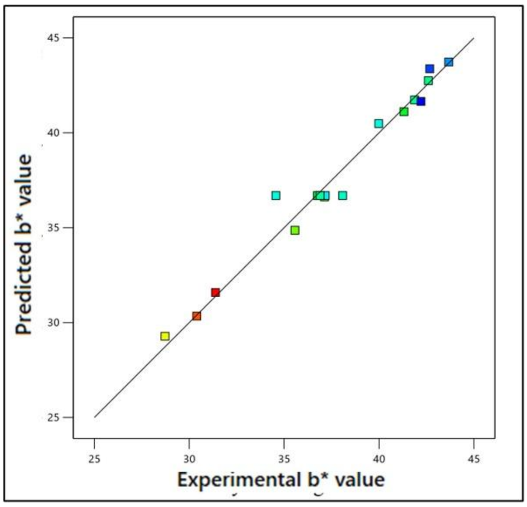


**Figure S5.** Comparison of experimental and RSM model-predicted values for the b* color value in vacuum microwave drying of Valencia oranges, displaying 1: 1 parity alignment within acceptable statistical prediction bands.

**Table S5.** Desirability-Based Numerical Optimization Solutions Generated by Design-Expert Software for Valencia Oranges in a Vacuum Microwave Dryer (Evaluated from the 17-Run BBD Experimental Model)

| Run | Power (kW) | Temperature (°C) | Slice Thickness (mm) | Drying Rate (kg $H_2O$/kg DM x s) | Rehydration Capacity (g/g) | b* value | Desirability | |
|---|---|---|---|---|---|---|---|---|
| **1** | **4.000** | **80.000** | **2.228** | **10.496** | **4.749** | **35.733** | **0.718** | **Selected** |
| 2 | 4.000 | 80.000 | 2.209 | 10.557 | 4.757 | 35.668 | 0.718 | |
| 3 | 4.000 | 80.000 | 2.176 | 10.664 | 4.772 | 35.554 | 0.717 | |
| 4 | 4.000 | 79.879 | 2.225 | 10.496 | 4.745 | 35.712 | 0.716 | |
| 5 | 4.000 | 79.962 | 2.159 | 10.713 | 4.777 | 35.495 | 0.716 | |
| 6 | 4.000 | 79.989 | 2.080 | 10.971 | 4.812 | 35.225 | 0.714 | |
| 7 | 2.000 | 80.000 | 2.201 | 9.381 | 5.031 | 35.012 | 0.713 | |
| 8 | 2.000 | 80.000 | 2.201 | 9.381 | 5.031 | 35.011 | 0.713 | |
| 9 | 4.000 | 80.000 | 2.037 | 11.115 | 4.831 | 35.074 | 0.712 | |
| 10 | 2.000 | 80.000 | 2.187 | 9.429 | 5.039 | 34.946 | 0.712 | |
| 11 | 4.000 | 80.000 | 2.026 | 11.153 | 4.836 | 35.033 | 0.712 | |
| 12 | 4.000 | 79.533 | 2.215 | 10.496 | 4.733 | 35.654 | 0.712 | |
| 13 | 2.000 | 79.997 | 2.228 | 9.295 | 5.016 | 35.128 | 0.711 | |
| 14 | 3.961 | 80.000 | 2.213 | 10.496 | 4.762 | 35.443 | 0.711 | |
| 15 | 2.000 | 79.911 | 2.196 | 9.391 | 5.031 | 34.949 | 0.711 | |
| 16 | 2.000 | 80.000 | 2.158 | 9.524 | 5.056 | 34.817 | 0.710 | |
| 17 | 3.986 | 80.000 | 2.023 | 11.144 | 4.839 | 34.940 | 0.709 | |
| 18 | 4.000 | 79.325 | 2.210 | 10.496 | 4.725 | 35.620 | 0.709 | |
| 19 | 4.000 | 79.048 | 2.202 | 10.496 | 4.716 | 35.574 | 0.705 | |
| 20 | 2.000 | 80.000 | 2.078 | 9.789 | 5.100 | 34.457 | 0.704 | |
| 21 | 3.954 | 80.000 | 2.026 | 11.095 | 4.843 | 34.756 | 0.703 | |
| 22 | 3.960 | 79.999 | 2.000 | 11.189 | 4.854 | 34.697 | 0.702 | |
| 23 | 2.063 | 80.000 | 2.188 | 9.423 | 5.031 | 34.613 | 0.699 | |
| 24 | 3.897 | 80.000 | 2.143 | 10.643 | 4.802 | 34.835 | 0.697 | |
| 25 | 2.128 | 80.000 | 2.174 | 9.469 | 5.031 | 34.231 | 0.685 | |
| 26 | 2.000 | 78.740 | 2.151 | 9.445 | 5.020 | 34.235 | 0.682 | |
| 27 | 2.000 | 80.000 | 2.610 | 8.126 | 4.812 | 36.758 | 0.682 | |
| 28 | 2.000 | 80.000 | 2.633 | 8.057 | 4.800 | 36.854 | 0.679 | |
| 29 | 3.828 | 80.000 | 2.019 | 10.971 | 4.868 | 34.017 | 0.677 | |
| 30 | 4.000 | 80.000 | 2.773 | 8.901 | 4.532 | 37.454 | 0.665 | |
| 31 | 2.000 | 80.000 | 2.815 | 7.547 | 4.708 | 37.587 | 0.658 | |
| 32 | 2.230 | 80.000 | 2.447 | 8.619 | 4.875 | 34.924 | 0.655 | |

| | | | | | | | |
|---|---|---|---|---|---|---|---|
| 33 | 4.000 | 73.817 | 2.039 | 10.496 | 4.574 | 34.859 | 0.647 |
| 34 | 2.000 | 80.000 | 2.928 | 7.245 | 4.652 | 38.027 | 0.643 |
| 35 | 4.000 | 65.954 | 2.000 | 9.680 | 4.411 | 35.271 | 0.596 |
| 36 | 4.000 | 60.000 | 2.000 | 8.847 | 4.390 | 36.325 | 0.596 |
| 37 | 4.000 | 60.341 | 2.000 | 8.897 | 4.389 | 36.249 | 0.595 |
| 38 | 4.000 | 60.454 | 2.000 | 8.914 | 4.388 | 36.225 | 0.595 |
| 39 | 4.000 | 61.514 | 2.000 | 9.069 | 4.386 | 36.005 | 0.593 |
| 40 | 4.000 | 64.102 | 2.000 | 9.432 | 4.394 | 35.541 | 0.592 |
| 41 | 4.000 | 61.817 | 2.000 | 9.112 | 4.386 | 35.945 | 0.592 |
| 42 | 4.000 | 63.777 | 2.000 | 9.387 | 4.392 | 35.593 | 0.592 |
| 43 | 4.000 | 62.443 | 2.000 | 9.201 | 4.387 | 35.826 | 0.592 |
| 44 | 4.000 | 63.373 | 2.000 | 9.331 | 4.390 | 35.661 | 0.592 |
| 45 | 3.957 | 61.328 | 2.000 | 8.994 | 4.400 | 35.632 | 0.584 |
| 46 | 4.000 | 60.000 | 3.196 | 5.880 | 4.218 | 40.538 | 0.510 |
| 47 | 2.000 | 68.348 | 2.808 | 6.608 | 4.614 | 33.533 | 0.485 |
| 48 | 4.000 | 60.000 | 6.000 | 3.579 | 4.270 | 46.067 | 0.392 |
| 49 | 4.000 | 60.111 | 5.997 | 3.586 | 4.264 | 46.031 | 0.391 |

**Table S6a.** T-test for the drying rate values obtained under optimal drying conditions in the vacuum microwave dryer for Valencia oranges (Statistical outputs in these tables are presented as raw software export logs. Please note that commas (,) serve as regional decimal separators and omit leading zeroes in accordance with software default formatting (e.g., ,17294 and ,114 correspond to international dot-decimal format 0.17294 and 0.114, respectively).

T-Test

**One-Sample Statistics**

| | N | Mean | Std. Deviation | Std. Error Mean |
|---|---|---|---|---|
| Drying rate | 3 | 10,5053 | ,42015 | ,24257 |

**One-Sample Test**

| | Test Value = 10.4962 | | | | | |
|---|---|---|---|---|---|---|
| | | | | | 95% Confidence Interval of the Difference | |
| | t | df | Sig. (2-tailed) | Mean Difference | Lower | Upper |
| Drying rate | ,038 | 2 | ,973 | ,00910 | -1,0346 | 1,0528 |

**One-Sample Effect Sizes**

| | | | | 95% Confidence Interval | |
|---|---|---|---|---|---|
| | | Standardizer[a] | Point Estimate | Lower | Upper |
| Drying rate | Cohen's d | ,42015 | ,022 | -1,113 | 1,151 |
| | Hedges' correction | ,74470 | ,012 | -,628 | ,649 |

a. The denominator used in estimating the effect sizes.
Cohen's d uses the sample standard deviation.
Hedges' correction uses the sample standard deviation, plus a correction factor.

**Table S6b.** T-test for the rehydration capacity values obtained under optimal drying conditions in the vacuum microwave dryer for Valencia oranges. Statistical outputs in these tables are presented as raw software export logs. Please note that commas (,) serve as regional decimal separators and omit leading zeroes in accordance with software default formatting (e.g., ,17294 and ,114 correspond to international dot-decimal format 0.17294 and 0.114, respectively).

T-Test

**One-Sample Statistics**

| | N | Mean | Std. Deviation | Std. Error Mean |
|---|---|---|---|---|
| Rehydration capacity | 3 | 4,4800 | ,17294 | ,09985 |

**One-Sample Test**

| | Test Value = 4.74923 | | | | | |
|---|---|---|---|---|---|---|
| | | | | | 95% Confidence Interval of the Difference | |
| | t | df | Sig. (2-tailed) | Mean Difference | Lower | Upper |
| Rehydration capacity | -2,696 | 2 | ,114 | -,26923 | -,6988 | ,1604 |

**One-Sample Effect Sizes**

| | | | | 95% Confidence Interval | |
|---|---|---|---|---|---|
| | | Standardizer[a] | Point Estimate | Lower | Upper |
| Rehydration capacity | Cohen's d | ,17294 | -1,557 | -3,320 | ,288 |
| | Hedges' correction | ,30653 | -,878 | -1,873 | ,163 |

a. The denominator used in estimating the effect sizes.
Cohen's d uses the sample standard deviation.
Hedges' correction uses the sample standard deviation, plus a correction factor.

**Table S6c.** T-test for the b* values obtained under optimal drying conditions in the vacuum microwave dryer for Valencia oranges. Statistical outputs in these tables are presented as raw software export logs. Please note that commas (,) serve as regional decimal separators and omit leading zeroes in accordance with software default formatting (e.g., ,17294 and ,114 correspond to international dot-decimal format 0.17294 and 0.114, respectively).

**T-Test**

**One-Sample Statistics**

| | N | Mean | Std. Deviation | Std. Error Mean |
|---|---|---|---|---|
| b* value | 3 | 35,8200 | ,62952 | ,36346 |

**One-Sample Test**

| | Test Value = 35.733 | | | | | |
|---|---|---|---|---|---|---|
| | | | | | 95% Confidence Interval of the Difference | |
| | t | df | Sig. (2-tailed) | Mean Difference | Lower | Upper |
| b* value | ,239 | 2 | ,833 | ,08700 | -1,4768 | 1,6508 |

**One-Sample Effect Sizes**

| | | Standardizer[a] | Point Estimate | 95% Confidence Interval Lower | 95% Confidence Interval Upper |
|---|---|---|---|---|---|
| b* value | Cohen's d | ,62952 | ,138 | -1,016 | 1,261 |
| | Hedges' correction | 1,11580 | ,078 | -,573 | ,712 |

a. The denominator used in estimating the effect sizes.
Cohen's d uses the sample standard deviation.
Hedges' correction uses the sample standard deviation, plus a correction factor.

**Table S7.** RSM-Based ANOVA Analysis of the Drying of Valencia Oranges in a Hot Air Dryer

| Source | Sum of Squares Error | df | Mean Square Error | F-value | p-value |
|---|---|---|---|---|---|
| **Drying Rate** | | | | | |
| Model | 5.69 | 9 | 0.6317 | 43.41 | < 0.0001 |
| Residual | 0.1019 | 7 | 0.0146 | | |
| Lack of Fit | 0.0422 | 3 | 0.0141 | 0.9418 | 0.4995 |
| Pure Error | 0.0597 | 4 | 0.0149 | | |
| Total | 5.79 | 16 | | | |
| $R^2 = 0.9824$ | | | | | |
| CV% = 9.36 | | | | | |
| **Rehydration Capacity** | | | | | |
| Model | 2.40 | 3 | 0.7998 | 23.01 | < 0.0001 |
| Residual | 0.4520 | 13 | 0.0348 | | |
| Lack of Fit | 0.3380 | 9 | 0.0376 | 1.32 | 0.4227 |
| Pure Error | 0.1140 | 4 | 0.0285 | | |
| Total | 2.85 | 16 | | | |
| $R^2 = 0.8415$ | | | | | |

CV% = 4.54

| | | | | | |
|---|---|---|---|---|---|
| **a* value** | | | | | |
| Model | 129.83 | 3 | 43.28 | 18.06 | < 0.0001 |
| Residual | 31.16 | 13 | 2.40 | | |
| Lack of Fit | 12.03 | 9 | 1.34 | 0.2796 | 0.9480 |
| Pure Error | 19.13 | 4 | 4.78 | | |
| Total | 160.99 | 16 | | | |
| $R^2$ = 0.8065 | | | | | |
| CV% = 22.14 | | | | | |

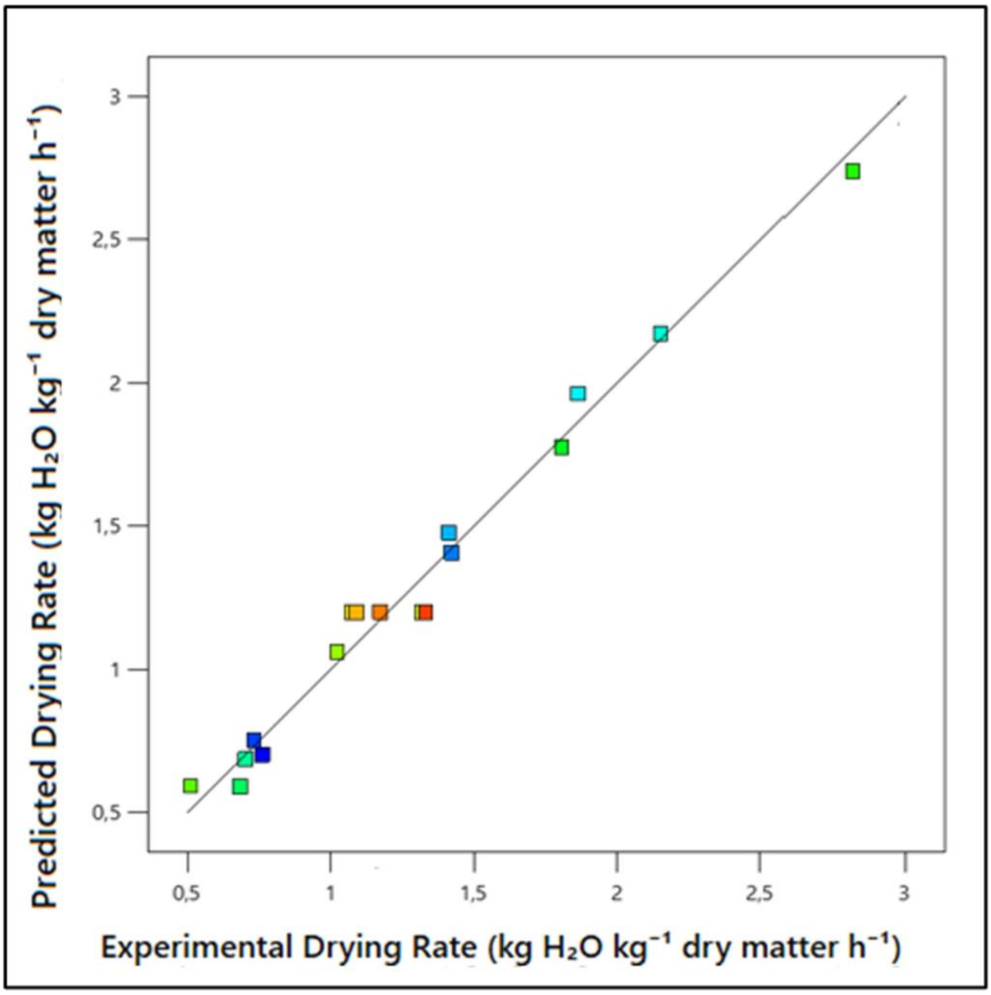


**Figure S6.** Comparison of experimental and RSM model-predicted values of drying rates in the hot air dryer for Valencia oranges, displaying 1:1 parity alignment within acceptable statistical prediction bands.

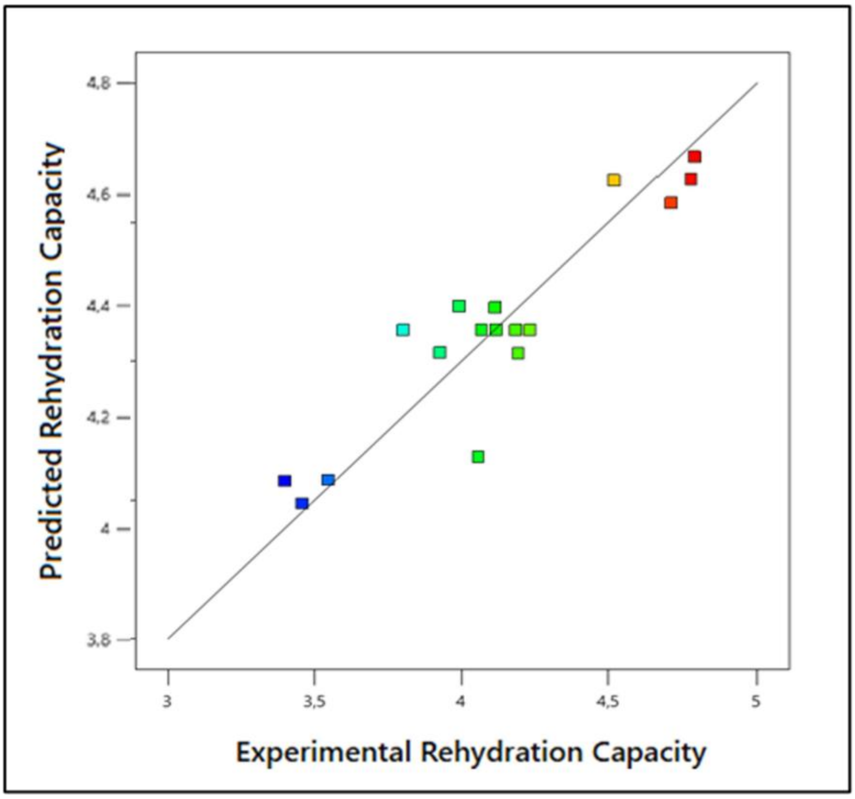


**Figure S7.** Comparison of experimental and RSM model-predicted values of rehydration capacity in the hot air dryer for Valencia oranges, displaying 1:1 parity alignment within acceptable statistical prediction bands.

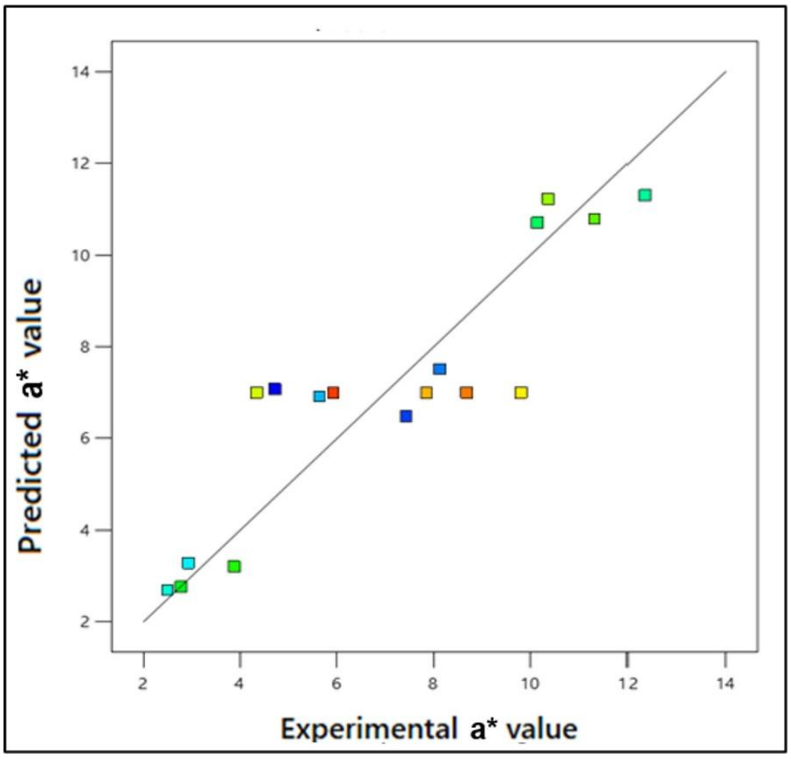

**Figure S8.** Comparison of experimental and RSM model-predicted values for the a* color value in the hot air dryer for Valencia oranges, displaying 1:1 parity alignment within acceptable statistical prediction bands.

**Table S8.** Desirability-Based Numerical Optimization Solutions Generated by Design-Expert Software for Valencia Oranges in a Hot Air Dryer (Evaluated from the 17-Run BBD Experimental Model)

| Solution | Air Speed (m/s) | Temperature (°C) | Slice Thickness (mm) | Drying Rate (kg $H_2O$/kg DM x s) | Rehydration Capacity (g/g) | b* value | Desirability | |
|---|---|---|---|---|---|---|---|---|
| **1** | **1.806** | **80.000** | **2.000** | **2.749** | **4.737** | **3.018** | **0.960** | **Selected** |
| 2 | 1.810 | 80.000 | 2.000 | 2.748 | 4.737 | 3.015 | 0.960 | |
| 3 | 1.798 | 80.000 | 2.000 | 2.750 | 4.737 | 3.023 | 0.960 | |
| 4 | 1.801 | 79.999 | 2.000 | 2.750 | 4.737 | 3.021 | 0.960 | |
| 5 | 1.822 | 80.000 | 2.000 | 2.746 | 4.737 | 3.008 | 0.960 | |
| 6 | 1.786 | 80.000 | 2.000 | 2.752 | 4.737 | 3.030 | 0.960 | |
| 7 | 1.829 | 80.000 | 2.000 | 2.745 | 4.737 | 3.004 | 0.960 | |
| 8 | 1.778 | 80.000 | 2.000 | 2.753 | 4.737 | 3.034 | 0.960 | |
| 9 | 1.758 | 80.000 | 2.000 | 2.755 | 4.737 | 3.046 | 0.960 | |
| 10 | 1.752 | 80.000 | 2.000 | 2.755 | 4.737 | 3.050 | 0.960 | |
| 11 | 1.872 | 80.000 | 2.000 | 2.736 | 4.737 | 2.978 | 0.960 | |
| 12 | 1.727 | 80.000 | 2.000 | 2.757 | 4.737 | 3.065 | 0.960 | |
| 13 | 1.715 | 80.000 | 2.000 | 2.758 | 4.737 | 3.072 | 0.960 | |
| 14 | 1.896 | 80.000 | 2.000 | 2.731 | 4.737 | 2.964 | 0.960 | |
| 15 | 1.839 | 79.937 | 2.001 | 2.739 | 4.736 | 2.999 | 0.959 | |
| 16 | 1.693 | 80.000 | 2.000 | 2.758 | 4.737 | 3.085 | 0.959 | |
| 17 | 1.783 | 79.858 | 2.000 | 2.744 | 4.736 | 3.029 | 0.959 | |
| 18 | 1.933 | 80.000 | 2.000 | 2.720 | 4.738 | 2.942 | 0.959 | |
| 19 | 1.803 | 79.836 | 2.000 | 2.740 | 4.736 | 3.016 | 0.959 | |
| 20 | 1.769 | 80.000 | 2.006 | 2.749 | 4.735 | 3.052 | 0.959 | |
| 21 | 1.947 | 80.000 | 2.000 | 2.716 | 4.738 | 2.934 | 0.958 | |
| 22 | 1.655 | 80.000 | 2.000 | 2.758 | 4.736 | 3.108 | 0.958 | |
| 23 | 1.967 | 80.000 | 2.000 | 2.709 | 4.738 | 2.922 | 0.958 | |
| 24 | 1.975 | 80.000 | 2.000 | 2.706 | 4.738 | 2.917 | 0.958 | |
| 25 | 1.629 | 80.000 | 2.000 | 2.757 | 4.736 | 3.123 | 0.958 | |
| 26 | 1.613 | 80.000 | 2.000 | 2.755 | 4.736 | 3.133 | 0.957 | |
| 27 | 1.607 | 80.000 | 2.000 | 2.755 | 4.736 | 3.137 | 0.957 | |

| | | | | | | | |
|---|---|---|---|---|---|---|---|
| 28 | 1.986 | 80.000 | 2.003 | 2.700 | 4.737 | 2.917 | 0.957 |
| 60 | 1.565 | 77.053 | 2.000 | 2.589 | 4.712 | 3.097 | 0.928 |
| 61 | 2.000 | 76.823 | 2.000 | 2.523 | 4.711 | 2.833 | 0.927 |
| 62 | 2.000 | 80.000 | 2.125 | 2.603 | 4.704 | 3.152 | 0.927 |
| 63 | 1.185 | 80.000 | 2.004 | 2.605 | 4.733 | 3.396 | 0.925 |
| 64 | 1.171 | 80.000 | 2.000 | 2.599 | 4.734 | 3.397 | 0.925 |
| 65 | 1.166 | 79.993 | 2.000 | 2.596 | 4.734 | 3.399 | 0.924 |
| 66 | 1.955 | 75.482 | 2.000 | 2.468 | 4.700 | 2.830 | 0.916 |
| 67 | 1.094 | 79.999 | 2.000 | 2.548 | 4.734 | 3.442 | 0.916 |
| 68 | 1.067 | 80.000 | 2.000 | 2.528 | 4.734 | 3.458 | 0.912 |
| 69 | 1.993 | 80.000 | 2.219 | 2.536 | 4.679 | 3.345 | 0.904 |
| 70 | 1.003 | 80.000 | 2.000 | 2.477 | 4.734 | 3.497 | 0.903 |
| 71 | 1.291 | 76.257 | 2.000 | 2.463 | 4.704 | 3.243 | 0.902 |
| 72 | 1.539 | 74.095 | 2.000 | 2.430 | 4.687 | 3.048 | 0.900 |
| 73 | 2.000 | 73.511 | 2.000 | 2.349 | 4.684 | 2.760 | 0.895 |
| 74 | 2.000 | 73.243 | 2.000 | 2.335 | 4.682 | 2.754 | 0.893 |
| 75 | 2.000 | 71.941 | 2.000 | 2.269 | 4.671 | 2.726 | 0.880 |
| 76 | 1.000 | 77.346 | 2.000 | 2.331 | 4.712 | 3.440 | 0.877 |
| 77 | 2.000 | 71.102 | 2.000 | 2.227 | 4.664 | 2.707 | 0.872 |
| 78 | 2.000 | 69.406 | 2.000 | 2.144 | 4.650 | 2.670 | 0.856 |
| 79 | 2.000 | 67.956 | 2.000 | 2.075 | 4.638 | 2.638 | 0.842 |
| 80 | 2.000 | 63.108 | 2.000 | 1.854 | 4.597 | 2.532 | 0.794 |
| 81 | 1.340 | 63.350 | 2.000 | 1.867 | 4.597 | 2.931 | 0.785 |
| 82 | 1.192 | 61.328 | 2.000 | 1.708 | 4.579 | 2.975 | 0.749 |

**Table S9a.** T-test for the drying rate values obtained under optimal drying conditions in the HAD for Valencia oranges Statistical outputs in these tables are presented as raw software export logs. Please note that commas (,) serve as regional decimal separators and omit leading zeroes in accordance with software default formatting (e.g., ,17294 and ,114 correspond to international dot-decimal format 0.17294 and 0.114, respectively).

T-Test

**One-Sample Statistics**

| | N | Mean | Std. Deviation | Std. Error Mean |
|---|---|---|---|---|
| Drying rate | 3 | 2,7251 | ,00348 | ,00201 |

**One-Sample Test**

| | Test Value = 2.74902 | | | | | |
|---|---|---|---|---|---|---|
| | | | | | 95% Confidence Interval of the Difference | |
| | t | df | Sig. (2-tailed) | Mean Difference | Lower | Upper |
| Drying rate | -11,915 | 2 | ,007 | -,02392 | -,0326 | -,0153 |

**One-Sample Effect Sizes**

| | | | | 95% Confidence Interval | |
|---|---|---|---|---|---|
| | | Standardizer[a] | Point Estimate | Lower | Upper |
| Drying rate | Cohen's d | ,00348 | -6,879 | -13,293 | -,941 |
| | Hedges' correction | ,00616 | -3,881 | -7,500 | -,531 |

a. The denominator used in estimating the effect sizes.
Cohen's d uses the sample standard deviation.
Hedges' correction uses the sample standard deviation, plus a correction factor.

**Table S9b.** T-test for the rehydration capacity values obtained under optimal drying conditions in the HAD for Valencia oranges. Statistical outputs in these tables are presented as raw software export logs. Please note that commas (,) serve as regional decimal separators and omit leading zeroes in accordance with software default formatting (e.g., ,17294 and ,114 correspond to international dot-decimal format 0.17294 and 0.114, respectively).

**T-Test**

**One-Sample Statistics**

| | N | Mean | Std. Deviation | Std. Error Mean |
|---|---|---|---|---|
| Rehydration capacity | 3 | 4,6307 | ,06850 | ,03955 |

**One-Sample Test**

| | Test Value = 4.73703 | | | | | |
|---|---|---|---|---|---|---|
| | | | | | 95% Confidence Interval of the Difference | |
| | t | df | Sig. (2-tailed) | Mean Difference | Lower | Upper |
| Rehydration capacity | -2,689 | 2 | ,115 | -,10636 | -,2765 | ,0638 |

**One-Sample Effect Sizes**

| | | | | 95% Confidence Interval | |
|---|---|---|---|---|---|
| | | Standardizer[a] | Point Estimate | Lower | Upper |
| Rehydration capacity | Cohen's d | ,06850 | -1,553 | -3,313 | ,290 |
| | Hedges' correction | ,12141 | -,876 | -1,869 | ,163 |

a. The denominator used in estimating the effect sizes.
Cohen's d uses the sample standard deviation.
Hedges' correction uses the sample standard deviation, plus a correction factor.

**Table S9c.** T-test for the a* values obtained under optimal drying conditions in the HAD for Valencia oranges. Statistical outputs in these tables are presented as raw software export logs. Please note that commas (,) serve as regional decimal separators and omit leading zeroes in accordance with software default formatting (e.g., ,17294 and ,114 correspond to international dot-decimal format 0.17294 and 0.114, respectively).

## T-Test

### One-Sample Statistics

| | N | Mean | Std. Deviation | Std. Error Mean |
|---|---|---|---|---|
| a* value | 3 | 3,0000 | ,36056 | ,20817 |

### One-Sample Test

| | Test Value = 3.01787 | | | | | |
|---|---|---|---|---|---|---|
| | | | | | 95% Confidence Interval of the Difference | |
| | t | df | Sig. (2-tailed) | Mean Difference | Lower | Upper |
| a* value | -,086 | 2 | ,939 | -,01787 | -,9135 | ,8778 |

### One-Sample Effect Sizes

| | | Standardizer[a] | Point Estimate | 95% Confidence Interval | |
|---|---|---|---|---|---|
| | | | | Lower | Upper |
| a* value | Cohen's d | ,36056 | -,050 | -1,176 | 1,089 |
| | Hedges' correction | ,63907 | -,028 | -,664 | ,614 |

a. The denominator used in estimating the effect sizes.
Cohen's d uses the sample standard deviation.
Hedges' correction uses the sample standard deviation, plus a correction factor.
